# Bidirectional regulation of desmosome hyperadhesion by keratin isotypes and desmosomal components

**DOI:** 10.1007/s00018-022-04244-y

**Published:** 2022-04-05

**Authors:** Fanny Büchau, Franziska Vielmuth, Jens Waschke, Thomas M. Magin

**Affiliations:** 1 Institute of Biology, Division of Cell and Developmental Biology, University of Leipzig, Philipp-Rosenthal-Straße 55, 04103 Leipzig, Germany; 2 Chair of Vegetative Anatomy, Institute of Anatomy, Faculty of Medicine, LMU Munich, Munich, Germany

**Keywords:** Desmosome, Atomic force microscopy, Keratins

## Abstract

Desmosomes are intercellular junctions which mediate cohesion and communication in tissues exposed to mechanical strain by tethering the intermediate filament cytoskeleton to the plasma membrane. While mature desmosomes are characterized by a hyperadhesive, Ca^2+^-independent state, they transiently loose this state during wound healing, pathogenesis and tissue regeneration. The mechanisms controlling the hyperadhesive state remain incompletely understood. Here, we show that upon Ca^2+^-induced keratinocyte differentiation, expression of keratin 17 (K17) prevents the formation of stable and hyperadhesive desmosomes, accompanied by a significant reduction of desmoplakin (DP), plakophilin-1 (PKP1), desmoglein-1 (Dsg1) and -3 (Dsg3) at intercellular cell borders. Atomic force microscopy revealed that both increased binding strength of desmoglein-3 molecules and amount of desmoglein-3 oligomers, known hallmarks of hyperadhesion, were reduced in K17- compared to K14-expressing cells. Importantly, overexpression of Dsg3 or DPII enhanced their localization at intercellular cell borders and increased the formation of Dsg3 oligomers, resulting in stable, hyperadhesive desmosomes despite the presence of K17. Notably, PKP1 was enriched in these desmosomes. Quantitative image analysis revealed that DPII overexpression contributed to desmosome hyperadhesion by increasing the abundance of K5/K17-positive keratin filaments in the proximity of desmosomes enriched in desmoglein-3. Thus, our data show that hyperadhesion can result from recruitment of keratin isotypes K5/K17 to desmosomes or from enhanced expression of DP and Dsg3 irrespective of keratin composition. The notion that hyperadhesive desmosomes failed to form in the absence of keratins underscores the essential role of keratins and suggest bidirectional control mechanisms at several levels.

**Supplementary Information:**

The online version contains supplementary material available at 10.1007/s00018-022-04244-y.

## Introduction

The resilience of the epidermis against physical stress depends largely on the keratin-desmosome complex which provides strong cell–cell adhesion [1]. In contrast to adherens junctions, desmosomes can occur in two distinct adhesive states. In the healthy epidermis, desmosomes adopt a Ca^2+^-independent, hyperadhesive state, which is important for stable intercellular adhesion and resistance to mechanical strain [2–4]. In contrast, during wound healing, desmosomes become Ca^2+^-dependent, resulting in weaker intercellular adhesion [3, 5, 6]. How the switch between the two adhesive states of desmosomes is regulated remains incompletely understood. One model suggests that hyperadhesion correlates with an ordered arrangement of the extracellular domains of the desmosomal cadherins, which can be identified by the presence of an electron-dense midline [2]. Furthermore, hyperadhesion correlated with reduced molecular mobility of desmosomal cadherins and DP [7] and enhanced binding strength and clustering of Dsg3 [8]. Moreover, elevated levels of PKP1 enhanced desmosome size and induced desmosome hyperadhesion by laterally interacting with DP, by modulating membrane availability and cluster formation of Dsg3 [9–13]. At the same time, increased levels of PKP3 prevent formation of hyperadhesive desmosomes [14] suggesting that desmosomal protein composition is one determinant regulating hyperadhesion. In addition, posttranslational modifications seem to partake in this regulation, exemplified by a PKCα-mediated switch from hyperadhesive to more dynamic Ca^2+^-dependent desmosomes during wounding in mouse skin [5, 14, 15]. At the wound margin, loss of hyperadhesive desmosomes was observed along with elevated expression of keratins K6, K16 and K17, which represent a wounding signature, and was accompanied by weaker contacts between keratins and the desmosomal plaque [1, 16].

We have previously established mouse keratinocyte cell lines which express a single type I keratin in a keratin type I null background (KtyI-/-) [17]. Expression of K17 (KtyI-/-mK17) stabilized primarily the endogenous type II keratin K6 and to a lesser extent K5 whereas keratinocytes expressing K14 (KtyI-/-mK14) as the only type I keratin contained higher levels of K5 than K6 protein [17]. Moreover, cells preferentially expressing the keratin pair K6/K17 showed less stable desmosomes whereas K5/K14 positive keratinocytes presented with stable desmosomes [17]. These and additional data strongly suggested that a threshold level of K5 is critical and sufficient for the maintenance of stable desmosomes with little internalization and degradation of desmosomal components. These findings raised the question to which extent composition and abundance of keratin filaments contribute to desmosomal hyperadhesion. Here, we report that KtyI-/-mK17 cells fail to establish hyperadhesive desmosomes, characterized by a significant reduction of DP, PKP1, Dsg1 and Dsg3 at intercellular cell borders. Overexpression of Dsg3 or DP restored the localization of DP and PKP1 at intercellular cell borders, led to increased formation of Dsg3 oligomers and the formation of stable, hyperadhesive desmosomes in K17 expressing cells. Image analysis indicated that in this setting, the level of K5/17 filaments was increased at desmosomes. The data suggest a model in which the organization and the composition of the keratin cytoskeleton together with that of desmosomal components determines the adhesive strength.

## Materials and methods

### Cell culture

Keratinocytes were cultured as previously described in [17–19]. Briefly, cells were cultivated in complete FAD media (0.05 mM CaCl_2_) on collagen I- (rat tail, Becton Dickinson) coated plates (coated for 30 min at 37 °C and then washed with 1 × PBS) at 32 °C, 5% CO_2._ Upon confluence, keratinocytes were split 1:2. For experiments, cells were seeded with a density of 4.2 × 10^4^ cells/cm^2^ and switched to 1.2 mM Ca^2+^-containing media for 24 h or 72 h one day after seeding. Respective K14 or K17 expressing keratinocytes stably expressing cell lines were generated by lentiviral transduction essentially as described earlier [20, 21]. To overexpress mouse Dsg3-GFP or DPII-GFP, cDNAs were cloned into lentiviral pLVX-Neo vector (Clontech) and stably expressing cell lines were generated by lentiviral transduction and subsequent FACS sorting of Dsg3- or DPII-GFP expressing cells.

### Immunostaining

Fixation of cells and staining was performed as previously described in [18]. Antibodies are listed in Supplementary table 1. Coverslips were placed into culture dishes prior to collagen coating. For keratin and DP staining, cells were fixed for 5 min in − 20 °C methanol, 30 s in –20 °C acetone. For PKP1 and PKP3 staining, cells were fixed for 10 min in − 20 °C methanol, and then permeabilized for 15 min in MT-extraction Buffer (100 mM PIPES, pH 6.9, 2 mM EDTA, 1 mM EGTA, 4 M Glycerin, 0.5% Triton X-100) and washed 3 × with TBS. For Dsg1 and Dsg3 staining, cells were fixed with ice-cold ethanol for 30 min, followed by 3 min ice-cold acetone. After a TBS wash, they were incubated overnight at 4 °C with primary antibodies diluted in TBS/1% BSA. Secondary antibodies at appropriate dilutions were applied and incubated for 60 min. Coverslips were mounted with Prolong Gold (Invitrogen).

### Immunofluorescence microscopy and data processing

Images for quantification of the keratin cytoskeleton were collected with a Zeiss LSM 780 confocal microscope equipped with 63×/1.46 NA oil immersion objective and Aryscan. The size of the pinhole was always set to 1 Airy unit. To quantify the fluorescence intensity, images were taken with a Zeiss Imager Z2 microscope equipped with ApoTome with a 40×/1.3 NA oil immersion objective and a AxioCam Mrm. Image’s with a pixel dimension of 0.161 µm × 0.161 µm were taken. Image analysis and processing were performed using Zen Software 2010 (Carl Zeiss, Inc.), Zen Blue Software (Carl Zeiss, Inc.), ImageJ and Photoshop CS4 (Adobe) software. LUT (lookup table; brightness) was adjusted using Photoshop.

### Western blotting

SDS-PAGE and Western blotting was performed as described [22]. Antibodies are listed in Supplementary table 1. In short: total proteins were extracted in SDS-PAGE sample buffer under repeated heating (95 °C). Separation of total protein extracts was carried out by standard procedures (6%, 8% and 10% SDS-PAGE). The immunoblotting was performed as described earlier (Vijayaraj et al., 2009).

### Surface biotinylation

3 × 10^5^ Cells, per well were seeded on a 6-well-plate. 24 h after plating, the medium was switched to high calcium medium for 24 h. Cells were washed 3 times with ice-cold PBS Mg/Ca and surface-labelled for 30 min with cell-impermeable EZ-Link Sulfo-NHS-SS-Biotin (Pierce) (1.5 mg/ml) following the manufacturer's instructions on ice. Cells were washed 3 times with PBS Mg/Ca containing 100 mM glycine for 20 min on ice to quench residual biotin. For cell surface measurements cells were lysed with 500 µl of cold RIPA buffer (10 mM Na_2_HPO_4_, 150 mM NaCl, 5 mM EDTA, 2 mM EGTA, 1% T-X-100, 0,1% SDS, 0,5% Sodium deoxycholate, pH 7.5), with 1 × protease and phosphatase inhibitor cocktails (Pierce). Lysates were incubated with 50 µl streptavidin beads (Pierce) for 1 h. Beads were washed 4 times with 800 µl cold RIPA buffer pH 7.5, with 1 × protease and phosphatase inhibitor cocktails. Biotinylated surface proteins were finally eluted by SDS-PAGE buffer and subsequently analyzed by western blotting. The amount of membrane-bound Dsg1 and Dsg3 was calculated as ratio between biotinylated protein to tubulin in the input fraction.

### Dispase assay

The assay was performed as described in [18, 23]. To test for hyperadhesive desmosomes, detached sheets were incubated in Low-Calcium Medium (LCM) with 3 mM EGTA for 1 h. Then, detached monolayers were exposed to mechanical stress by orbital rotation (15 rounds in 30secs) in 4 ml PBS in a 15-ml Falcon tube. For quantification an image of the complete six-well was taken and the fragments of each six-well with a growth area of 9.5cm^2^ were counted.

### Purification of recombinant desmoglein Fc-constructs

Dsg1- and Dsg3-Fc constructs containing the full extracellular domain of the respective Dsg were stably expressed in Chinese Hamster Ovary cells (CHO cells) and purified using protein A agarose (Life Technologies). Purification was performed as described elsewhere in detail [24, 25].

### Atomic force microscopy measurements (AFM)

A NanoWizard® 3 AFM or Nanowizard® 4 AFM (both JPK Instruments, Berlin, Germany) mounted on inverted optical microscopes (Carl Zeiss, Jena, Germany or IX73 Olympus, Hamburg, Germany, respectively) was used throughout all experiments. Regions along intercellular cell borders were chosen by usage of an optical image acquired with a 63 × objective and topography overview images acquired in JPK-QI-mode. All measurements were accomplished at 37 °C in cell culture medium containing 1.2 mM Ca^2+^. For adhesion measurements pyramidal-shaped D-Tips of Si3N4 MLCT cantilevers (Bruker, Mannheim, Germany) with a nominal spring constant of 0.03 N/m and tip radius of 20 nm were functionalized with Dsg1- or Dsg3-Fc constructs as described before using a flexible heterobifunctional acetal-polyethyleneglycol (synthetized by the Hermann Gruber Lab, Institute of Biophysics, Linz, Austria) [26]. All adhesion measurements on living murine keratinocytes were obtained in force mapping mode. Small areas (6 × 2 µm) along intercellular cell borders were chosen and 1200 pixel with each pixel representing one force-distance curve were acquired. The experimental setup was described in detail before and is shown in Fig. S1 a,b. Briefly, the following parameters were used: setpoint: 0.5nN, Z-length: 1.5 µm, pulling speed: 10 µm/s and resting contact time: 0.1 s. For characterization of molecule distribution at the cell border areas a distribution coefficient was calculated as described before [27, 28] with higher values indicating an increased binding frequency along intercellular cell borders compared to surrounding cell surface.

### Chemical crosslinking experiments

For investigation of protein oligomerization of desmosomal cadherins, the membrane-impermeable crosslinker ethylene glycol bis (sulfosuccinimidyl succinate) (Sulfo-EGS) (Pierce Biotechnology, Rockford, USA) was used. The assay was performed as previously described [11, 29]. Briefly, 3 × 10^5^ cells per well were seeded on a 6-well-plate. 24 h after plating, the medium was switched to high calcium medium for 24 or 72 h. Cells were washed 3 times with ice-cold PBS Mg/Ca and incubated with 2 mM Sulfo-EGS in PBS for 30 min at room temperature. TBS at a concentration of 50 mM was added to stop the reaction. Detection of crosslinked products was performed by western blotting.

### Cytoskeletal fractionation

For the preparation of keratin-enriched cytoskeletal protein fractions, cultured cells were washed twice with ice-cold PBS and lysed in ice-cold low-salt buffer with RNase (5 µg/ml), DNase (2.5 µg/ml) and 1 × protease- and phosphatase inhibitor cocktail. Cell residues were sheared with a Dounce homogenizer, incubated for 5 min on ice and centrifuged for 10 min at 5000 × g (4 °C). The supernatant, representing the soluble fraction, was collected in a new tube and Laemmli sample buffer was added and the sample was boiled for 5 min at 95 °C. The pellet was resuspended in ice-cold high-salt buffer with RNase (5 µg/ml), DNase (2.5 µg/ml) and 1 × protease- and phosphatase inhibitor cocktail and sheared again with the Dounce homogenizer and incubated for 10 min. This procedure was repeated 3 times. After centrifugation for 10 min at 15.000 × *g* (4 °C), the pellet, representing the keratin-enriched fraction was resuspended in Laemmli sample buffer and boiled for 5 min at 95 °C.

### Quantification of immunostainings

To quantify the accumulation of Dsg3, PKP1 and PKP3 at intercellular cell borders, fluorescence intensities were measured in segments of equal length and width covering the cytoplasm and bicellular contacts as illustrated in Fig. S2 a. The mean junctional fluorescence intensities were divided by the corresponding mean cytoplasmic values for each line scan, for a total of > 500 individual measurements. All calculated ratios are shown as boxplots displaying the first to third quartile with the full range of variation. To depict the mean distribution of PKP1, DSP and PKP3 along the line scans, fluorescence intensities along the line scans were normalized to the corresponding mean cytoplasmic value. Statistical significances between the cell lines was calculated using the Mann–Whitney-Rank-Sum test. To assess the irregularity of DP membrane staining, an irregularity score was calculated. The fluorescence intensities were measured in segments of equal length and width covering the bicellular border as illustrated in Fig S2 b. The intensity profile of the border of a cell was plotted using Zen Blue (Zeiss), and the corresponding average fluorescence intensity x was calculated. Then, for each pixel along the cell membrane, it was determined whether that pixel was brighter than the average x, in which case this pixel gave a contribution of its intensity. Minus the average intensity. This was done for all pixels along the cell membrane and the average deviation was calculated. Finally, the result was divided by the average fluorescence intensity × to normalize for differently strong staining. In that way, the irregularity score solely is a measure for how dominant intensity differences are along the cell membrane, with an irregularity score of 0 meaning that all pixels have the same intensity. Analysis of the keratin cytoskeleton was performed using ImageJ. Junction density and branching was estimated by skeletonizing the image and measuring the number of junctions using the Skeleton plug-in. For this a region of interest of equal length and with covering the cell border and the cytoplasm directly underneath were used. In a first step, a threshold image was generated using the same parameters for each cell and each cell type. Afterwards the ImageJ plug-in Skeleton was used to skeletonize the image and to analyze keratin density by calculating the number of bundles or the number of bundle junctions per area (Size of ROI).

### Statistical analysis

Statistical significance was determined by two-tailed *t *tests. When equal variance test failed, Wilcoxon–Mann–Whitney Rank Sum test was run (*p* 0.05 = *; *p* 0.01 = **; *p* 0.005 = ***).

## Results

### Formation of hyperadhesive desmosomes is impaired in K17 expressing cells

**Fig. 1:**
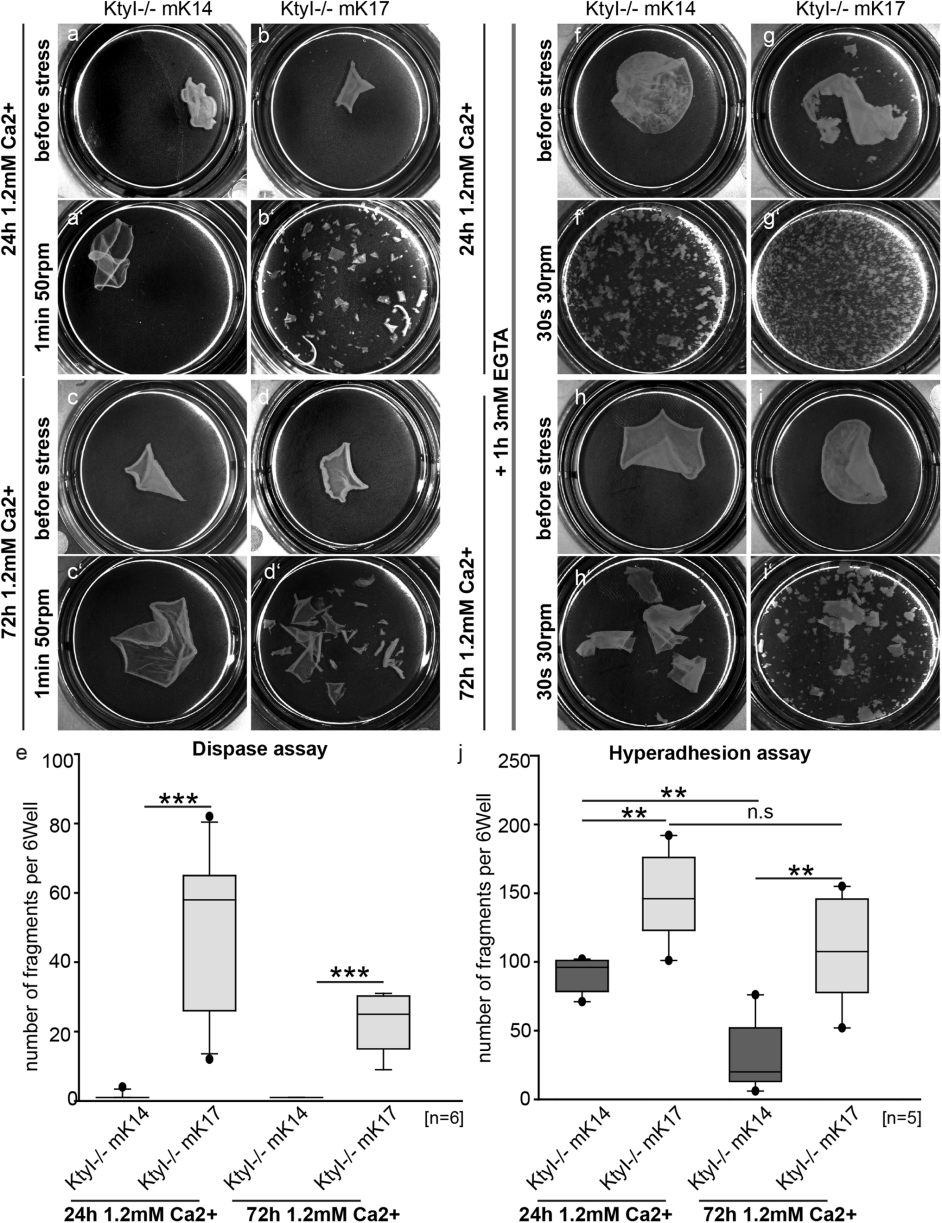
K17 expression reduces desmosome stability and impairs formation of hyperadhesive desmosomes. **a–e** Dispase assay after 24 h or 72 h in medium with 1.2 mM Ca^2+^, showing decreased epithelial sheet stability in KtyI-/-mK17 cells. **a**‴′ For quantification, numbers of fragments in a six-well were counted. *N* = 6; ns not significant; *p < 0.05, **p < 0.01, ***p < 0.001, Student’s *t *test. **f–j** Hyperadhesion assay after 24 h or 72 h in medium with 1.2 mM Ca^2+^, showing a highly impaired formation of Ca^2+^-independent desmosomes in K17 expressioning cells. **i**′ For quantification, numbers of fragments in a six-well were counted. N = 5; ns not significant; *p < 0.05, **p < 0.01, ***p < 0.001, Student’s *t *test

In cell culture, desmosome formation depends on extracellular Ca^2+^. Subsequently, desmosomes acquire a hyperadhesive state during maturation. To investigate whether desmosomes in K17 (KtyI-/-mK17)-compared to K14-expressing keratinocytes (KtyI-/-mK14) become hyperadhesive, a mechanical cell dissociation assay was performed with cells grown for 24 h and 72 h in high Ca^2+^ medium. In this assay, cell sheets were lifted with dispase, treated with 3 mM EGTA for 1 h and then subjected to mechanical stress. This hyperadhesion dispase assay showed a time- dependent significant decrease of fragment numbers in KtyI-/-mK14 cells indicative of desmosome maturation, whereas more and smaller fragments were detected in KtyI-/- mK17 cells, indicating that expression of K17 prevented the formation of hyperadhesive desmosomes (Fig. 1).

### Keratin isotypes regulate localization and abundance of desmosomal cadherins Dsg1 and Dsg3

**Fig. 2:**
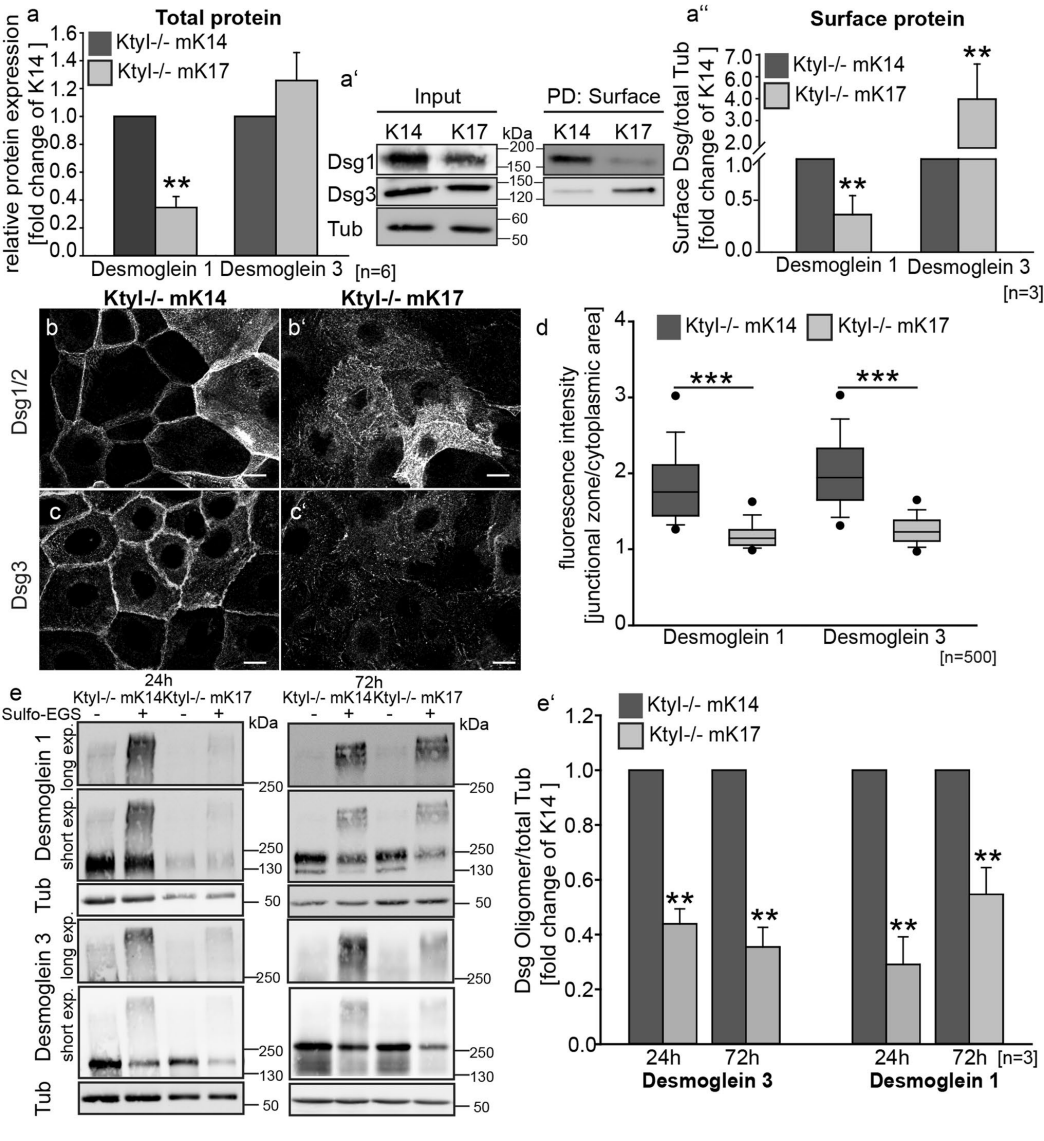
Keratin isotype-dependent abundance and distribution of Dsg1 and Dsg3 (**a–a**′) Western Blot analysis of Dsg1 and Dsg3 levels in total protein lysates from KtyI-/-K14- and KtyI-/-mK17 cells 24 h after Ca^2+^ switch, showing decreased total Dsg1- and very slightly increased total Dsg3 levels in K17 expressing cells, compared to KtyI-/-mK14 cells. **a** Quantification of relative Dsg1 and Dsg3 protein levels from 6 different experiments. Tubulin was used as loading control, (mean ± SEM, *n* = 6, ns not significant; *p < 0.05, **p < 0.01, ***p < 0.001, Student’s *t *test.). **a**′–**a**″ Surface biotinylation assay revealed decreased Dsg1- and increased Dsg3 levels in K17 expressing cells at the cell surface. **a**″ Quantification of Dsg1- and Dsg3-levels at the cell-surface. Tubulin was used as loading control (mean ± SEM, *n* = 3, ns not significant; *p < 0.05, **p < 0.01, ***p < 0.001, Student’s *t *test.). **b–d** Staining of Dsg1/2 or Dsg3 after switching to medium with 1.2 mM Ca^2+^ demonstrates a patchy upregulation of Dsg1/2 in some cells of the monolayer in K14 and K17 expressing cells, in contrast to a homogenous expression of Dsg3 in all cells of the monolayer. Dsg1/2 and Dsg3 localized at cell borders in KtyI-/-K14 cells, whereas increased extradesmosomal localization and reduced border staining in KtyI-/-mK17 cells was apparent. Representative confocal sections are shown. Scale bars: 10 µm. **d** For quantification, the ratio of fluorescence intensity of Dsg1/2 and Dsg3 at junctions and in the cytoplasm was calculated using intensity profiles. 500 cell–cell borders of 3 independent experiments were analyzed, ns not significant; *p < 0.05, **p < 0.01, ***p < 0.001, Man–Whitney–Rank Sum test. **e–e**′ Chemical crosslinking experiments with Sulfo-EGS show a significant reduction of Dsg1 and Dsg3 clusters at the cell surface of KtyI-/-mK17 cells in comparison to KtyI-/-mK14 cells. **e**′ Quantification of relative amounts of Dsg1 and Dsg3 oligomers. Tubulin was used as loading control (mean ± SEM, *n* = 3, ns not significant; *p < 0.05, **p < 0.01, ***p < 0.001, Student’s *t *test)

Since according to the current model a conformational shift in Dsg and desmocollin (Dsc) extracellular domains correlates with the acquisition of hyperadhesion [30, 31], we investigated the role of Dsg’s in mediating adhesive strength in K14 compared to K17 cells. Western blot analysis revealed that total and cell surface-associated Dsg1 levels were significantly reduced in K17-expressing cells (Fig. 2a–a″), which is in line with our previous observation [17]. In contrast Dsg3 was significantly increased at the cell surface after 24 h in high Ca^2+^ medium (Fig. 2a-a″). Immunostaining using Dsg1/2 and Dsg3-specific antibodies showed that in KtyI-/- mK14 cells, desmosomal cadherins accumulated at the intercellular cell border, whereas in K17-expressing keratinocytes Dsg’s were reduced at the intercellular border and became increased at extradesmosomal locations (Fig. 2b-d, Suppl. Figure 1 a–d″). Clustering of desmosomal cadherins is one mechanism involved in the formation of hyperadhesive desmosomes [6, 8, 11]. To investigate desmosomal protein clustering by biochemical means, cross-linking of desmogleins was performed using the membrane-impermeable crosslinker Sulfo-EGS, followed by western blotting do detect desmoglein oligomers, as described before [8, 11, 29]. For quantification of oligomers, irrespective of the difference in Dsg total protein amount, we calculated the amount of Dsg oligomers relative to tubulin, serving as loading control. This showed that after 24 h and even more so after 72 h, the amount of Dsg1 and Dsg3 oligomers was significantly reduced in KtyI-/-mK17 compared to KtyI-/-mK14 cells (Fig. 2e-e′) indicating reduced protein clustering. These data indicate that although Dsg3, in contrast to Dsg1, was increased at the entire cell surface of KtyI-/-mK17 cells, lateral border localization and less clusters of both desmosomal cadherins exist in KtyI-/-mK17 cells compared to KtyI-/-mK14 cells.

### Adhesive properties of Dsg3 but not of Dsg1 are altered in K17-expressing keratinocytes compared to KtyI-/-mK14 cells

**Fig. 3:**
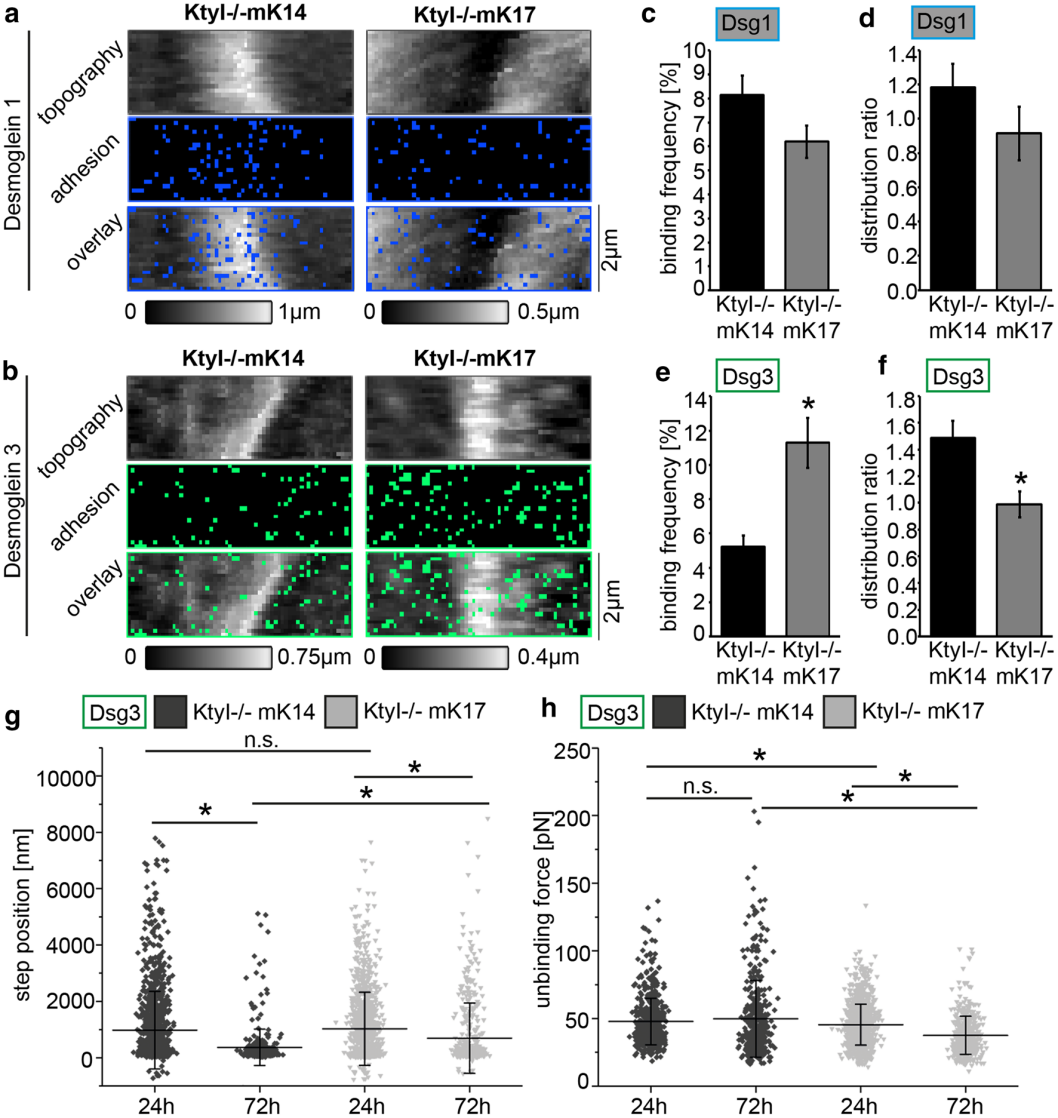
Dsg3 single molecule binding properties differ in KtyI -/- mK14 and mK17 cells. **a**, **b** AFM adhesion maps at small areas along cell borders of respective murine keratinocytes. In topography panels cell borders are distinguishable as elevated structures. Each pixel represents one force-distance curve and every blue pixel a specific Dsg1 binding event (**a**) or green pixel representing a Dsg3 binding event (**b**). Binding frequency (**c**) and distribution ratio (**d**) of Dsg1 were slightly reduced in KtyI-/-mK17 compared to KtyI-/-mK14 keratinocytes. Binding frequency of Dsg3 (**e**) was significantly increased in KtyI-/-mK17 compared to KtyI-/-mK14 cells whereas distribution ratio (**f**) was significantly decreased. Quantification of step position (**g**) and unbinding force (**h**) for Dsg3 in cells differentiated for 24 or 72 h in high Ca^2+^ medium. Step position decreased from 24 to 72 h in KtyI-/-mK14 and KtyI-/-mK17 keratinocytes. Further, step position was higher in KtyI-/-mK17 compared to KtyI-/-mK14 cells after 72 h. Unbinding force was slightly increased after 72 h in KtyI-/-mK14 but was diminished in KtyI-/-mK17 cells compared to 24 h of differentiation. In addition, unbinding force was lower in KtyI-/-mK17 compared to KtyI-/-mK14 cells both after 24 and 72 h. **a**, **b** Representatives of *n* = 3–4. **c**–**h** *n* = 3–4, 2400 force-distance curves/experiment from > 2 independent coating procedures. *p < 0.05, One-Way-ANOVA, Bonferroni, average ± SEM (**c**–**f**) ± SD (**g**, **h**)

Formation of hyperadhesive desmosomes is accompanied by altered binding properties of single desmosomal cadherins [8]. Thus, AFM force measurements were performed to investigate whether the keratin isotype affects binding properties of single desmosomal cadherins. AFM tips were functionalized with recombinant Dsg3-Fc or Dsg1-Fc comprising the complete extracellular domains of the respective protein (Suppl. Figure 1 e–f), and cells were examined after 24 and 72 h in high Ca^2+^ medium. AFM topography images showed elevated lateral intercellular cell borders in both cell lines (Fig. 3a–b). AFM adhesion maps were recorded at lateral intercellular cell borders and at the apical surface next to the lateral border, shown by blue and green rectangles (Fig. 3a–b). After 24 h in high Ca^2+^ medium, the binding frequency of Dsg1 was slightly reduced, in contrast to a strong increase of the Dsg3 binding frequency in KtyI-/-mK17 cells (Fig. 3c, e). Next, we analyzed the distribution ratio between Dsg binding events located at cell junctions and at non-junctional areas. While Dsg1 and Dsg3 binding events were more abundant along the cell border areas of KtyI-/-mK14 cells, the distribution in KtyI-/-mK17 cells was more uniform. This difference was significant for Dsg3 only (Fig. 3d, f). In addition, the step position of Dsg1 and Dsg3 interactions was measured. For this, the distance from the contact point to the point at which the bond ruptured was calculated. A higher step position indicates a so-called membrane tether [32] and supposedly serves as an indirect measure for cytoskeletal anchorage [11, 33, 34]. The step position for Dsg1-interactions decreased from 24 to 72 h in both cell lines in a similar fashion, indicating that the cytoskeletal anchorage of the measured Dsg1 molecules increased over time independently of the keratin isotype (Suppl. Figure 1 g). The step position for Dsg3-interactions also decreased over time in both cell lines, but was significantly higher in KtyI-/-mK17 cells, compared to KtyI-/-mK14 cells after 72 h in high Ca^2+^ medium, suggesting that the cytoskeletal anchorage of the measured Dsg3 was weaker in KtyI-/-mK17 cells (Fig. 3 g). Furthermore, we determined the strength of Dsg1 and Dsg3 single molecule interactions, referred to as unbinding force. Interestingly, after 24 h in high Ca^2+^, the unbinding force of Dsg1 was very similar between KtyI-/-mK14 and KtyI-/-K17 cells (Suppl. Figure 1 h), but was significantly decreased for Dsg3 in KtyI-/-mK17 compared to KtyI-/-mK14 cells (Fig. 3 h). To analyze whether the binding strength changes when desmosomes become hyperadhesive, the unbinding force for Dsg3 was measured 24 h and 72 h after the Ca^2+^-switch. The differences between unbinding forces did not reach statistical significance but the unbinding force increased slightly from 24 to 72 h in KtyI-/-mK14 cells, whereas in K17-expressing keratinocytes it significantly decreased further from 24 to 72 h (Fig. 3h). These data indicate that the keratin isotype influences the binding properties of Dsg3 but not of Dsg1. The reduced binding strength of Dsg3 in the KtyI-/-K17 cells correlated with the impaired formation of hyperadhesive desmosomes.

### Altered localization of desmosomal plaque proteins and different keratin organization contribute to the adhesion defect in KtyI-/-mK17 cells

**Fig. 4:**
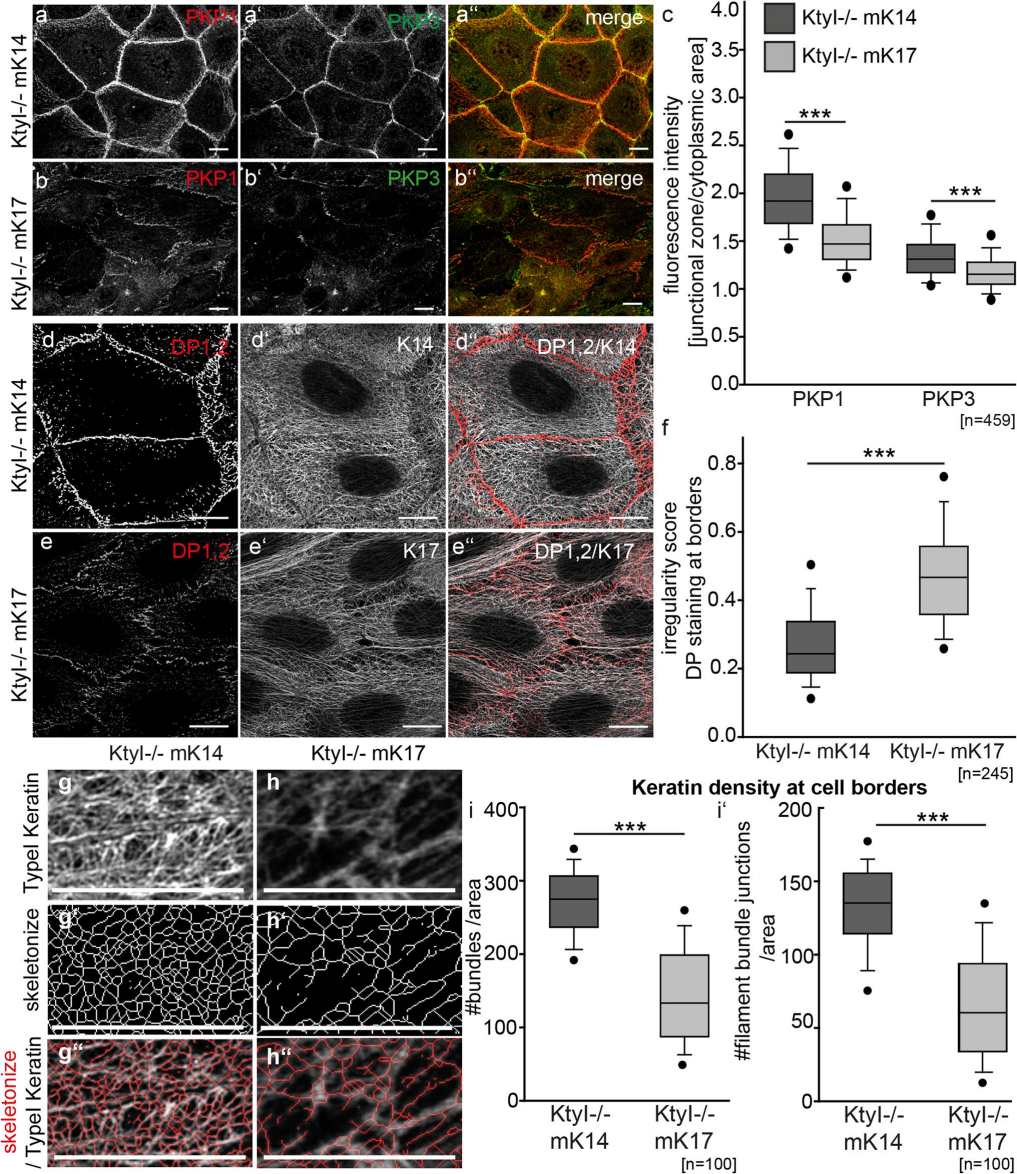
Reduction of desmosomal plaque proteins and keratin network density in KtyI-/-mK17 cells. **a–b**″ Representative confocal sections of PKP1 and PKP3 staining 24 h after Ca^2+^ switch show reduction of PKP1 and PKP3 at cell borders in KtyI-/-mK17 compared to KtyI-/-mK14 cells. Scale bar = 10 µm. **c** For quantification, the ratio of fluorescence intensity of PKP1 and 3 at junctions and in the cytoplasm was calculated using intensity profiles. 459 cell–cell borders of 3 independent experiments were analyzed, ns not significant; *p < 0.05, **p < 0.01, ***p < 0.001, Man–Whitney–Rank Sum test. **d–e**″ Co-staining of DP and K14 or K17 revealed an irregularly distributed DP signal at cell borders and a loosely organized keratin cytoskeleton at KtyI-/-mK17 cell borders. Scale bar = 10 µm. Images show representative confocal sections. **f** Quantification of the irregularity score for DP at borders. 245 cell–cell borders of 3 independent experiments were analyzed, ns not significant; *p < 0.05, **p < 0.01, ***p < 0.001, Man-Whitney-Rank Sum test. **g**–**h** Representative confocal Images of staining of K14 or K17. **g**–**h**″ Representative skeletonized image of keratin staining at cell borders of KtyI-/-mK14 and KtyI-/-mK17 cells using the ImageJ PlugIn Skeleton. **i–i**′ Keratin density at cell borders was calculated with the number of filament bundles or number of filament junctions divided by the area, using the ImageJ PlugIn Skeleton. 100 ROIs of 3 independent experiments were analyzed, ns not significant; *p < 0.05, **p < 0.01, ***p < 0.001, Man–Whitney–Rank Sum test

To understand how a particular keratin isotype affects localization, clustering and adhesive properties of desmosomal cadherins, we analyzed desmosomal plaque proteins in more detail. Others have shown that PKP1 is important for the formation of hyperadhesive desmosomes by promoting Dsg3 clustering and modulating its binding strength [8, 11, 14]. We have previously reported that PKP1 and 3 were slightly reduced at the total protein level [17]. Based on immunostaining, we found that the lateral border localization of PKP1 and 3 was significantly reduced in KtyI-/-mK17 compared to KtyI-/-mK14 cells (Fig. 4a-c). Knockout studies showed that DP is also important for the clustering of desmosomal cadherins, without affecting adhesive properties of single desmosomal cadherins [35]. Consistent with our previous data, fluorescent intensities of DP at the cell border revealed an irregular distribution of DP along the cell junctions in KtyI-/-mK17 cells ([17], Fig. 4d–f). These data implicate that the keratin isotype controls the localization of DP and PKP at the membrane, which might regulate the clustering and adhesive properties of desmosomal cadherins, preferentially of Dsg3. Both DP, as well as the keratin isotype impact on the organization of the keratin cytoskeleton [36, 37]. To address this, we compared the organization of the keratin cytoskeleton in the proximity of desmosomes in KtyI-/-mK14 and KtyI-/-mK17 cells. To determine the keratin density at the cell border, K14, K17 and DP were immunostained and analysed with the LSM 780 equipped with an Airyscan™ detector delivering super-resolution images (> 140 nm laterally, 400 nm axially). Image analysis using the ImageJ-PlugIn Skeleton showed that the number of filaments/bundles per area as well as the filament junctions per area were significantly reduced at intercellular cell borders of KtyI-/-mK17 cells, indicating a reduced network density in KtyI-/-mK17 cells, compared to KtyI-/-mK14 cells (Fig. 4g–i’, Suppl. Figure 3e-f’).

### Overexpression of DPII or Dsg3 rescued desmosome stability in KtyI-/-mK17 cells

**Fig. 5:**
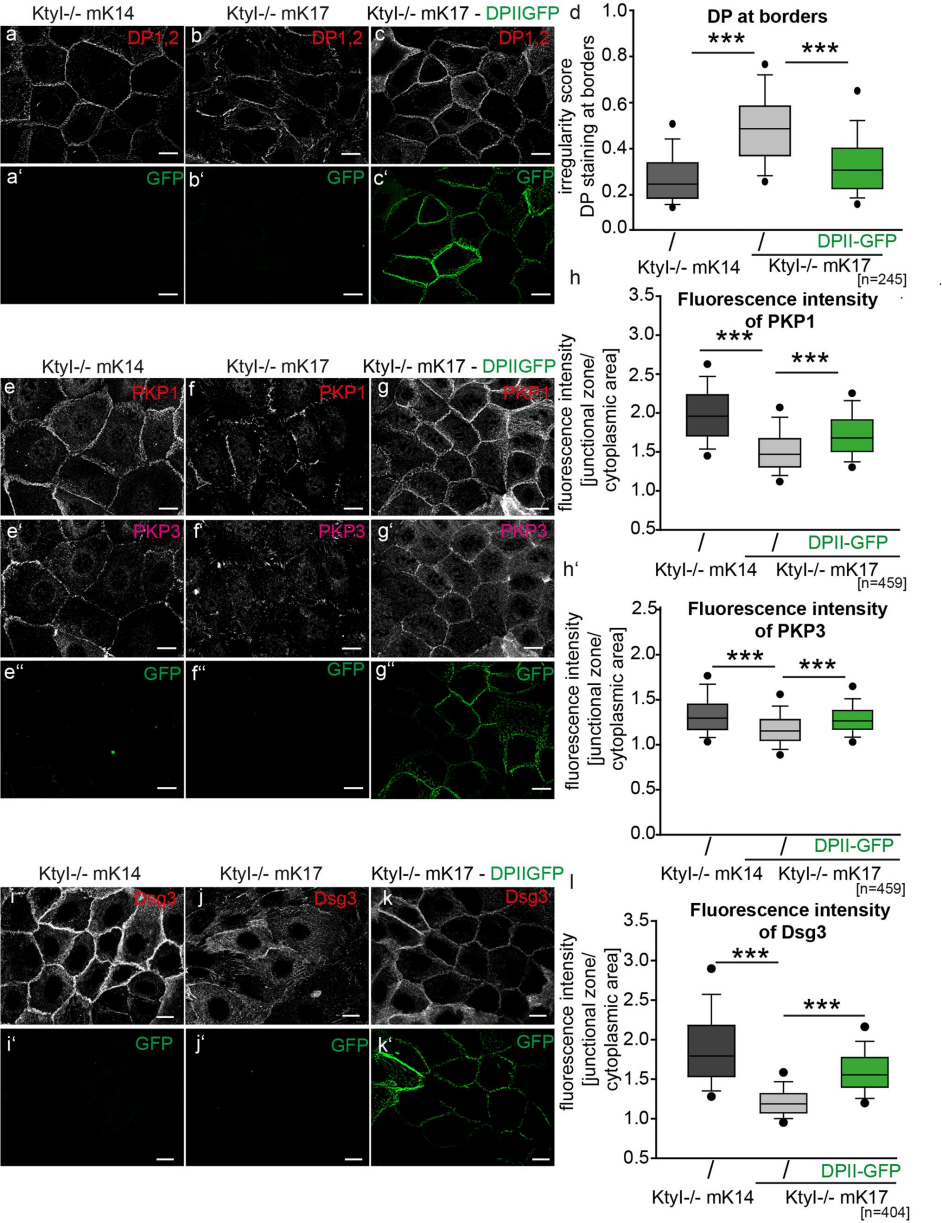
DPII-GFP overexpression in KtyI-/-mK17 cells affects localization of plaque proteins and of Dsg3. **a–c**′ Representative confocal sections of DP staining in KtyI-/-mK14, KtyI-/-mK17 cells, and KtyI-/-mK17 cells overexpressing DPIIGFP, 24 h after Ca^2+^-induced junction formation. Scale bar = 10 µm. **d** Quantification of the irregularity score for DP at borders showed that the irregular DP localization at junctions in KtyI-/-mK17 cells was restored after DPII overexpression. 245 cell–cell borders of 3 independent experiments were analyzed, ns not significant; *p < 0.05, **p < 0.01, ***p < 0.001, Mann–Whitney Rank Sum test. **e**–**g**″ Immunostaining of PKP1 and PKP3 in KtyI-/-mK14, KtyI-/-mK17 cells, and KtyI-/-mK17 cells overexpressing DPIIGFP 24 h after Ca-induced junction formation. Images show representative confocal sections. Scale bar = 10 µm. **h**–**h**′ For quantification, the ratio of fluorescence intensity of PKP1 and 3 at junctions and in the cytoplasm was calculated using intensity profiles. 459 cell–cell borders of 3 independent experiments were analyzed, ns not significant; *p < 0.05, **p < 0.01, ***p < 0.001, Man–Whitney–Rank Sum test. **i**–**k**′ Representative confocal sections of Dsg3 staining. Scale bar = 10 µm. **l** Quantification of the relative fluorescence intensities of Dsg3 at cell borders. 404 cell–cell borders of 3 independent experiments were analyzed, ns not significant; *p < 0.05, **p < 0.01, ***p < 0.001, Man–Whitney–Rank Sum test

To examine whether the reduced amount of DP and its irregular distribution at the cell border were responsible for the adhesive defects observed in KtyI-/-mK17 cells, we overexpressed DPII-GFP. Immunostaining showed that DPII-GFP localized correctly at lateral cell borders. In addition, the overall amount of DP at intercellular cell borders was significantly increased in KtyI-/-mK17 cells overexpressing DPII-GFP (Fig. 5a–d). Western blotting showed that the total amount of endogenous DPI and II increased after overexpression of DPII-GFP (Suppl. Figure 2 c–c’’). In contrast, after overexpression of DPII-GFP in keratinocytes lacking the entire keratin cytoskeleton (KtyI-/-) DPII-GFP failed to accumulate at lateral intercellular cell borders to the same extent and did not affect the localization of endogenous DP (Suppl. Figure 5 e–h). In addition, immunostaining showed that DPII-overexpression in KtyI-/-mK17 cells promoted a shift of other desmosomal proteins from the cytoplasm to intercellular cell borders. In particular, a significant amount of Dsg3, PKP1 and PKP3 molecules was localized correctly at the intercellular cell borders of KtyI-/-mK17 cells overexpressing DPII-GFP (Fig. 5e–l). Importantly, restoration of DP levels by overexpression of DPII-GFP in KtyI-/-mK17 cells significantly increased the abundance of Dsg3 oligomers at the cell surface, as detected by crosslinking (Suppl. Figure 2d–d’).

**Fig. 6:**
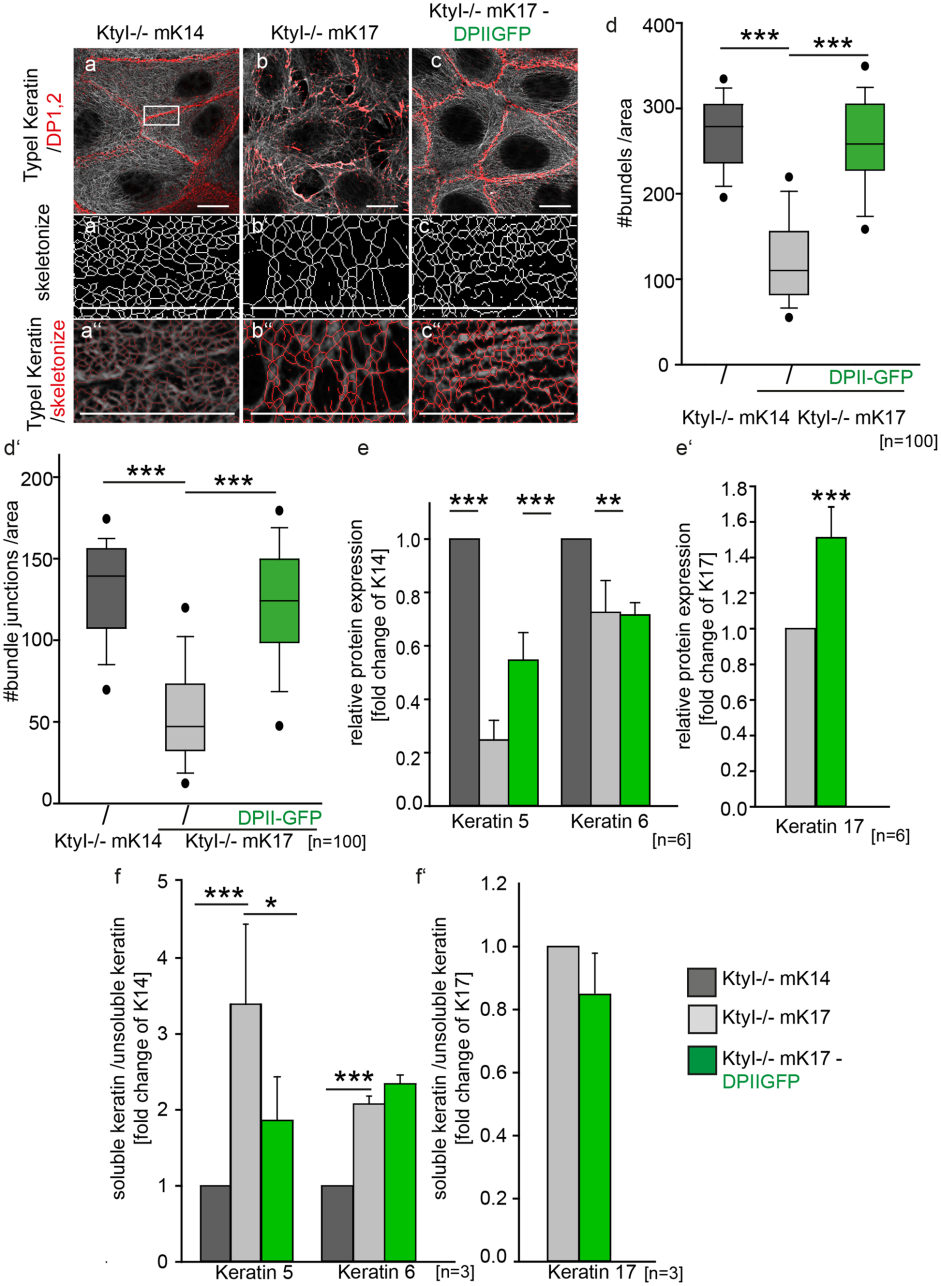
DPII-GFP overexpression in KtyI-/-mK17 cells increases keratin network density and stabilizes epithelial sheets. **a**–**c** Representative confocal section of DP/keratin staining of KtyI-/-mK14 and KtyI-/-mK17 cells and KtyI-/-mK17 cells overexpressing DPII-GFP. **a**′–**c**′ Skeletonized image of keratin staining at cell borders using the ImageJ PlugIn Skeleton. **a″**–**c**″ Overlay of skeletonized image with confocal image of K14 or K17 staining. Scale bar = 10 µm. **d**–**d**′ The keratin density at cell borders was calculated with the number of filament bundles or number of filament junctions divided by the area using the ImageJ PlugIn skeleton. 100 ROIs of 3 independent experiments were analyzed, ns not significant; *p < 0.05, **p < 0.01, ***p < 0.001, Man–Whitney–Rank Sum test. **e**–**e**′ Quantification of western blots of total protein lysates show that DPII overexpression increased the total amount of K5 and K17, but did not change the abundance of K6 in KtyI-/-mK17 cells. GAPDH was used as loading control (mean ± SEM, *n* = 6, ns not significant; *p < 0.05, **p < 0.01, ***p < 0.001, Student’s *t*-test.). **f**–**f**′ Cytoskeletal fractionation and subsequent WB of soluble and insoluble fractions revealed significantly decreased solubility of K5 in KtyI-/-mK17 cells overexpressing DPII-GFP, whereas K6 and K17 solubility remained largely unaltered. For quantification, the ratio of soluble and insoluble keratins was calculated. (mean ± SEM, *n* = 3, ns not significant; *p < 0.05, **p < 0.01, ***p < 0.001, Student’s *t*-test)

**Fig. 7:**
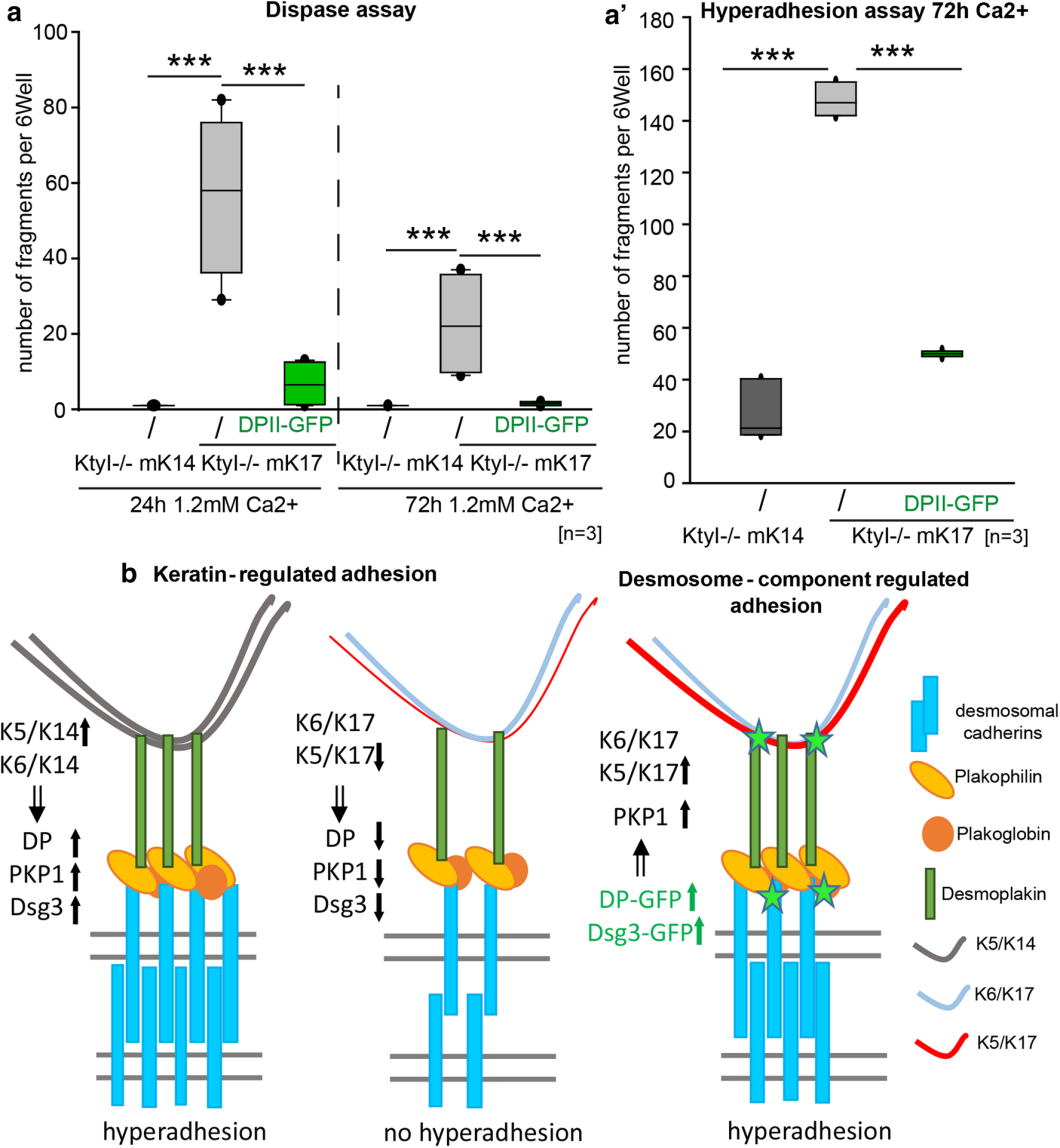
Manipulation of keratins or desmosomal components regulate stability of desmosomes and keratin organization. **a–a**′ Quantification of dispase and hyperadhesion assays showed that sheet stability and formation of hyperadhesive desmosomes was rescued by overexpression of DPII-GFP in KtyI-/-mK17 cells. *N* = 3; ns not significant; *p < 0.05, **p < 0.01, ***p < 0.001, Student’s *t*-test. **b** Schematic model of Cross-talk between the keratin cytoskeleton and desmosomes. In KtyI-/-mK14 cells a dense network of mainly K5/K14 and to a lesser extent K6/K14 filaments correlate with desmosomes enriched in desmoplakin and PKP1 and abundant clusters of desmosomal cadherins. This goes along with the formation of stable, hyperadhesive desmosomes. In contrast, KtyI-/-mK17 cells form instable, Ca^2+^-dependent desmosomes only. We showed that a loosely organized keratin network and reduced amount of K5-filaments lead to reduced amounts of DP and PKPs at the cell border. The reduced clustering of desmosomal cadherins could be a direct consequence of the reduction of DP and PKPs or may be regulated indirectly by the keratin isotype. Stability and formation of hyperadhesive desmosomes were restored by overexpression of DPII-GFP in the presence of K17. Furthermore, our data indicate that DPII-GFP overexpression in KtyI-/-mK17 cells recruited PKP1 and increased cluster formation of Dsg3 directly or indirectly through PKP1. In addition, DPII-GFP overexpression increased keratin network density and the amount of filamentous K5. In line with this, Dsg3-GFP overexpression stabilized PKP1 and DP at cell borders and rescued the formation of stable, hyperadhesive desmosomes in the presence of K17

Given that Dsg3 clusters at the cell-border were found to be important for the formation of hyperadhesive desmosomes [8], we overexpressed Dsg3-GFP in KtyI-/-mK17 cells. As a consequence, the cell border localization of Dsg3 and the plaque proteins DP, PKP1 and PKP3 was significantly increased in KtyI-/-mK17 cells upon expression of Dsg3-GFP (Suppl.3 and Suppl. Figure 4a–e, Suppl. Fig. 5a–d). In agreement, this correlated with an increased stability of desmosomes in the dispase assay (Suppl. 6a–g). Moreover, Dsg3-GFP overexpression restored the formation of hyperadhesive desmosomes in KtyI-/-mK17 cells significantly (Suppl. 6h–k). Thus, K17 per se does not preclude the formation of hyperadhesive desmosomes. We hypothesized that keratin organization next to desmosomes and overall keratin composition represents a determinant of desmosome hyperadhesion. To address this, the keratin cytoskeleton was immunostained and its network organization in DPII-GFP-overexpressing cells was analyzed by the ImageJ plugin Skeleton. In fact, overexpression of DPII-GFP increased the keratin density at the desmosome significantly (Fig. 6a–d′). In particular, co-staining of K5/K17- and K6/K17- positive filaments indicated that the amount of K5-positive filaments was reduced in KtyI-/-mK17 cells relative to K6/K17-positive filaments (Suppl. Figure 8a–h′). Most notably, DPII-GFP overexpression in KtyI-/-mK17 cells increased the abundance of K5/K17 filaments (Suppl. Figure 8e–f′). In support, western blotting revealed an increase in total levels of K5 and K17 in DPII-overexpressing cells whereas K6 remained constant (Fig. 6e–e′, Suppl. Figure 78 i). In this setting, the insoluble, filamentous fraction of K5/K17 was increased whereas K6 solubility remained unaltered (Fig. 6f–f′ and Suppl. Figure 8j). Collectively, this suggested a specific increase in K5/K17 over K6/K17 filaments. To investigate whether DPII-GFP overexpression restored desmosome stability and the formation of hyperadhesive desmosomes, we performed dispase and hyperadhesion dispase assays. After overexpression of DPII-GFP in KtyI-/-mK17 cells, the number of fragments strongly decreased at 24 h and 72 h. This was accompanied by a significant increase in hyperadhesive desmosomes in those cells (Fig. 7a–a′ and Suppl. Figure 7a–i′). In line with our previous data, this strongly indicates that the level of K5 is crucial for the formation of stable desmosomes. We conclude that overexpression of DPII-GFP preferentially stabilizes K5/K17 filaments to promote the formation of stable, hyperadhesive desmosomes (Fig. 7b).

## Discussion

This study reveals the existence of a bidirectional crosstalk between keratins and desmosomes. We found that the specific keratin-isotype composition and the density of the keratin network in the proximity of desmosomes are major determinants of desmosome composition and adhesive properties of the desmosomal cadherin Dsg3. Conversely, altering desmosome composition by overexpression of Dsg3 or DP altered keratin organization and isotype composition and promoted the formation of hyperadhesive desmosomes, even in the presence of K17 (Fig. 7b).

### Impact of the keratin cytoskeleton on the formation of hyperadhesive desmosomes

Desmosomes have the unique ability to acquire two different adhesive states. A weaker, Ca^2+^-dependent state during junction assembly and wound healing and a strong Ca^2+^-independent state, described as hyperadhesive, upon maturation [5]. Here we show that the formation of hyperadhesive desmosomes is strongly impaired in cells which express the wound-healing keratin K17. Previous studies indicated that maturation of desmosomes requires the clustering of the desmosomal cadherin Dsg3 and is accompanied by enhanced binding strength of Dsg3 molecules [8, 11]. Interestingly, Western blotting and AFM measurements revealed that Dsg1 was significantly reduced at the surface of KtyI-/-mK17 cells in contrast to Dsg3, which was increased. However, despite the elevated Dsg3 levels at the entire cell surface of KtyI-/-mK17 cells, immunostaining and crosslinking, indicative of cell surface-located Dsg3 clusters, revealed that the amount of Dsg1 and Dsg3 were strongly reduced at lateral intercellular cell borders and that clustering of both desmosomal cadherins was significantly decreased in KtyI-/-mK17 cells. Consistently, AFM measurements with Dsg1- and Dsg3-coated cantilevers show that the binding strength of Dsg1 was not affected by the keratin-isotype investigated here. In contrast, the formation of hyperadhesive desmosomes in KtyI-/-mK14 cells correlated with an increase in binding strength of Dsg3 whereas in KtyI-/-mK17 cells the binding strength of Dsg3 was significantly reduced. In line with this, the cytoskeletal anchorage of single Dsg1 molecules was similar in KtyI-/-mK14 and KtyI-/-mK17 cells, in contrast to a weaker anchorage of Dgs3 molecules after 72 h in KtyI-/-mK17 cells compared to KtyI-/-mK14 cells.

It was reported previously that in the absence of the entire keratin cytoskeleton, the binding strength of Dsg3 was reduced [28, 38]. Now, our data show for the first time that the density of the keratin-network and keratin isotype composition have a distinct impact on localization and binding properties of desmosomal cadherins, features which correlate with the formation of hyperadhesive desmosomes. Increased keratin network density together with the different keratin isotype composition might reduce Dsg mobility and enhance clustering of desmosomal cadherins in KtyI-/-mK14 cells in contrast to KtyI-/-mK17 cells. Indeed, clustering of desmosomal cadherins is important for proper adhesive function [39, 40]. Previous studies reported that membrane availability and clustering of Dsg’s strongly depended on the plaque proteins PKP1, 3 and the cytolinker DP [11, 35]. Loss of DP strongly reduced clustering of desmosomal cadherins but had no effect on the binding properties of single Dsg molecules [35]. In this setting, loss of PKP1 reduced membrane availability, cluster formation and the binding strength of Dsg3 significantly [11]. As PKP1 and DP are strongly reduced in KtyI-/-mK17 cells, these data indicate that the keratin cytoskeleton regulates Dsg localization, cluster formation and binding strength through PKP1 and DP. At present, it remains unknown whether the keratin network organization and the keratin isotype composition of the cytoskeleton regulate DP and PKP1 localization at the desmosomal plaque via direct protein interactions or through the differential regulation of signalling pathways. It is well established that the localization of DP and PKP1 at the desmosomal plaque is partially regulated by phosphorylation of both proteins [41, 42]. Given the complexity of keratin phosphorylation [43, 44] it is conceivable that keratins regulate phosphorylation of DP and PKP1 in an isotype-specific manner to control the accumulation of both proteins at the plaque.

### Desmosome protein composition affects keratin organization and adhesion

Interestingly, overexpression of Dsg3 or DP in KtyI-/-mK17 cells restored the ability of K17 expressing cells to form hyperadhesive desmosomes to a considerable extent. We found that overexpression of Dsg3 recruited PKP1 and DP to cell junctions in KtyI-/-mK17 cells and promoted increased clustering of Dsg3 at the cell surface. In addition, overexpression of DP also stabilized PKP1 at intercellular cell borders and increased clustering of Dsg3.

Consistent with previous reports [8, 11, 14, 35], our data strongly support the hypothesis that the abundance of clustered Dsg3 at the cell surface, which depends on sufficient levels of PKP1 and DP, is crucial for the formation of stable and hyperadhesive desmosomes. Remarkably, overexpression of DP not only affected the composition and adhesive properties of the desmosomal plaque in K17 cells but had an impact on the composition and organization of the keratin cytoskeleton. Whether the observed increase in keratin network density and increased levels of K5 in K17-expressing cells results from desmosomal or extra-desmosomal DP, remains an important issue for future experiments. Previously, the loss of DP was shown to strongly reduce anchorage of keratins at desmosomes [36, 45]. Here, we report that overexpression of DPII-GFP caused accumulation of DP at lateral intercellular cell borders of KtyI-/-mK17 cells, accompanied by the reorganization of the keratin network, in particular of K5/17 filaments, close to desmosomes. Based on our previous data that a threshold level of K5 is critical and sufficient to maintain stable desmosomes in keratinocytes [17], we postulate that the abundance of K5/17-containing filaments promotes the formation of hyperadhesive desmosomes in KtyI-/-mK17 cells overexpressing DPII-GFP. The mechanisms by which elevated levels of DP preferentially recruit K5/K17 have to be investigated in the future. Clearly, the failure of DP overexpression to maintain stable desmosomes in the absence of keratins underlines the importance of the keratin cytoskeleton for the formation of stable, hyperadhesive desmosomes.

### Supplementary Information

Below is the link to the electronic supplementary material.

Supplementary file1 (DOCX 5008 kb)

## Data Availability

Most of the data generated or analysed during this study are included in this published article and its supplementary information files. Additional datasets generated for quantification of the immunostainings for example are available from the corresponding author on reasonable request.
