# Supplementary material for: Bidirectional regulation of desmosome hyperadhesion by keratin isotypes and desmosomal components
Source: Cell Mol Life Sci. 2022 Apr 5;79(5):223. doi: 10.1007/s00018-022-04244-y (PMC8983532; doi:10.1007/s00018-022-04244-y)
Supplement: Supplementary file 1 — Supplementary file1 (DOCX 5008 kb) [file 18_2022_4244_MOESM1_ESM.docx]

**Supplementary Information**

**Bidirectional regulation of desmosome hyperadhesion by keratin isotypes and desmosomal components**

Fanny Büchau^1*^, Franziska Vielmuth^2^, Jens Waschke^2^, Thomas M. Magin^1^

^1^ Institute of Biology, Division of Cell and Developmental Biology, University of Leipzig, Leipzig, Germany

^2^ Chair of Vegetative Anatomy, Institute of Anatomy, Faculty of Medicine, LMU Munich, Munich, Germany

.

**Supplementary Table 1 Antibodies**

| **Primary antibodies** | **dilution IF/WB** | **host** | **source** |
| --- | --- | --- | --- |
| Desmoplakin1,2 | 1:150/1:1.000 | guinea pig | PSL Heidelberg |
| Desmoplakin (Il-5F)  (detects isoforms 1+2) | 1:150 (IF) | mouse | D.Garrod, Manchester (UK) |
| Desmoglein 3.1  (detects isoforms 1+2)  Cat. No. 61002 | 1:10 /1:100 | mouse | Progen, Heidelberg |
| Desmoglein 1  Cat. No. ab124798 | 1:1000 (WB) | rabbit | Abcam |
| Desmoglein 3 (AK18) | 1:400/1:2000 | mouse | Biozol |
| Plakophilin 1 | 1:1000 | rabbit | PSL Heidelberg |
| Plakohilin 3 | 1:500 | guinea pig | PSL Heidelberg |
| GAPDH  Cat. No. 2118 | 1:2000 (WB) | rabbit | Cell signaling |
| Keratin 14 | 1:100/1:25.000 | rabbit | PSL Heidelberg |
| Keratin 17 | 1:100/1:50.000 | rabbit | PSL Heidelberg |
| Keratin 5 | 1:100/ 1:30.000 | rabbit | PSL Heidelberg |
| Keratin 6 | 1:400 /1:50.000 | rabbit | PSL Heidelberg |
| Alpha-Tubulin  Cat. No. T9026 | 1:12.000 (WB) | mouse | Sigma Aldrich |
| **Secondary antibodies** | **dilution** | **host** | **source** |
| anti-mouse-, anti-rabbit-, anti-rat-, anti-guinea-pig-DL488, 549 | 1:800 | donkey | Dianova, Hamburg |
| anti-mouse-, anti-rabbit-, anti-rat-, anti-guinea-pig-DL649 | 1:400 | donkey | Dianova, Hamburg |
| anti-mouse-, anti-rabbit-HRP | 1:20.000 | donkey | Dianova, Hamburg |

**
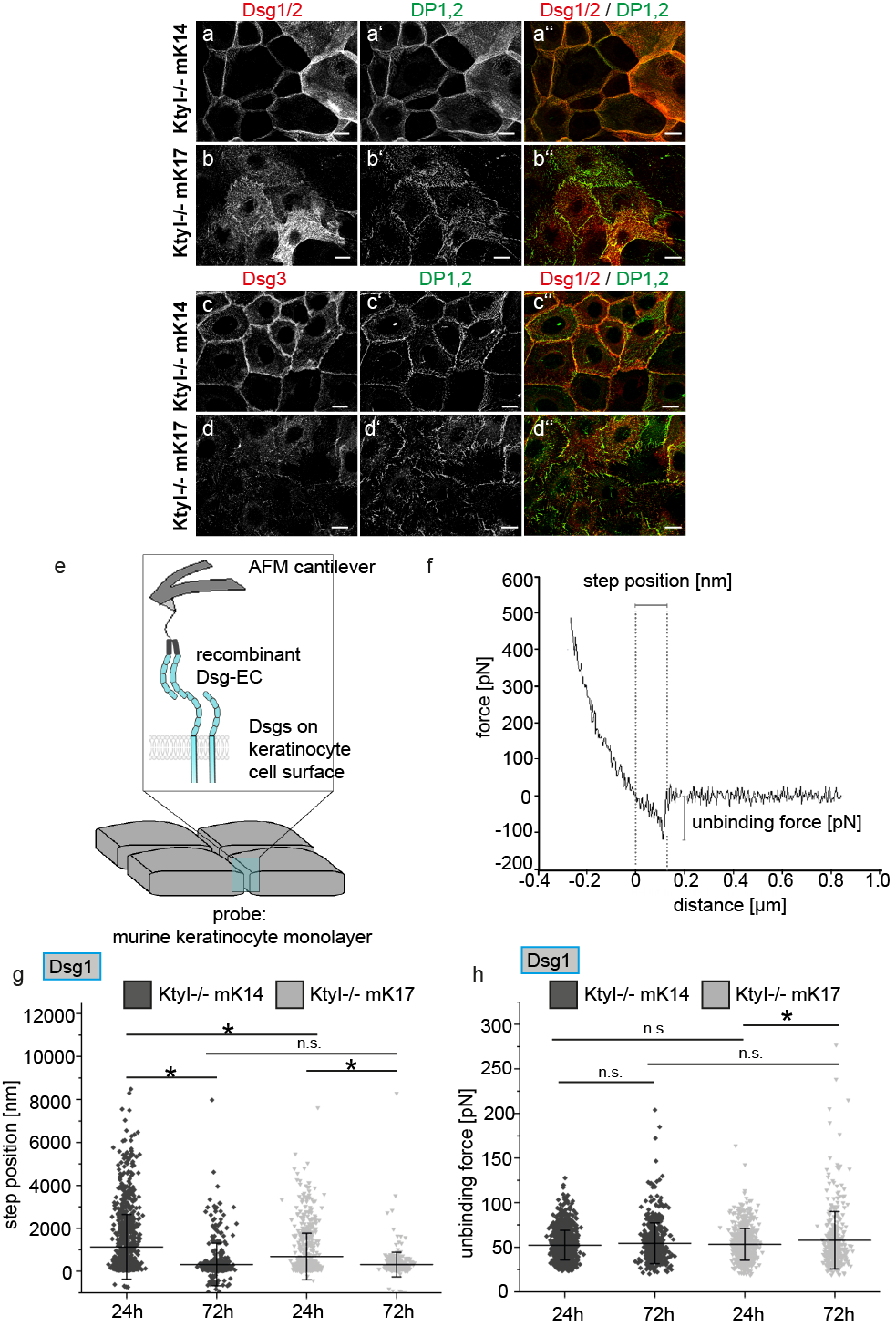
**

**Supplementary Figure 1 Dsg1 single molecule binding properties are equal in KtyI -/- mK14 and mK17 cells** (a-b’’) Representative confocal section of co-staining of Dsg1/2 and DP. (c-d’’) Representative confocal section of co-staining of Dsg1/2 and DP. Scale bar= 10µm. (e) Schematic of AFM measurements. A sharp tip on a flexible cantilever was functionalized with recombinant full-length extracellular domains (EC) of Dsg1 or 3 respectively. During adhesion measurements the cantilever was repetitively lowered to and retracted from the probe to allow specific single molecule interactions during contacting the probe (here: living murine keratinocytes). (f) Example of a force-distance curve with a specific binding event. During each approach-retract cycle a force-distance curve is acquired which can be analyzed with regards to occurrence of specific binding events and their biophysical properties such as step position and unbinding force. Quantification of step position (g) and unbinding force (h) for Dsg1 in cells differentiated for 24 or 72h in high Ca^2+^ medium. Step position is diminished from 24 to 72h in KtyI-/-mK14 and KtyI-/-mK17 keratinocytes and is smaller in KtyI-/-mK17 compared to KtyI-/-mK14 cells after 24h. Unbinding forces were comparable in both cell lines. N=3-4, 2400 force-distance curves/experiment from >2 independent coating procedures. *p<0.05, One-Way-ANOVA, Bonferoni, average +/- SD.

**
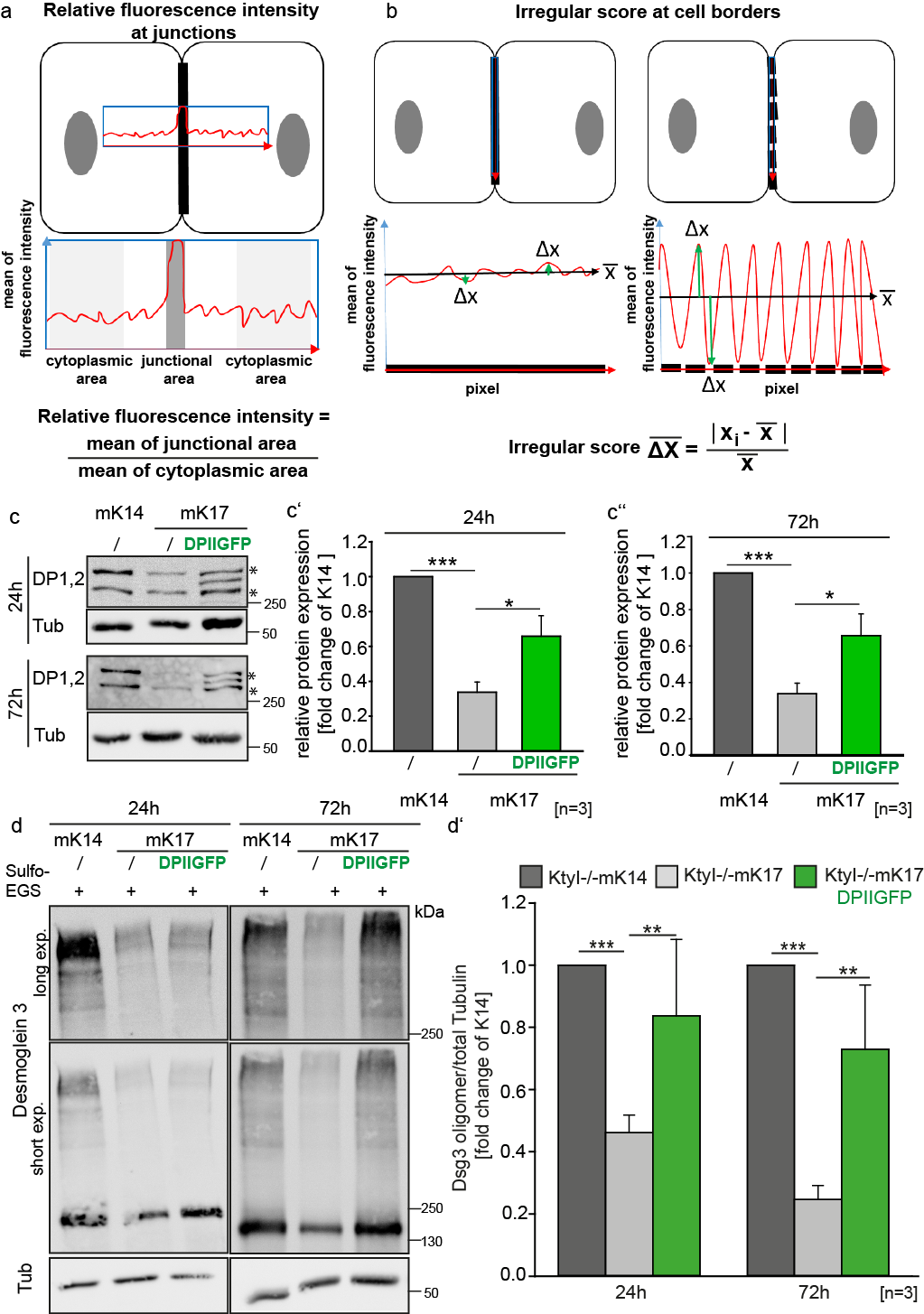
**

**Supplementary Figure 2 Quantification of fluorescence images and crosslinking analysis of Dsg3** (a) Schematic drawing explaining how the relative signal intensity of Dsg3, PKP1 and PKP3 at cell-cell junctions was calculated. (b) Schematic drawing explaining how the irregular score of DP staining at cell-cell junctions was calculated. (c-c’’) Western Blot analysis of DP1,2 levels in total protein lysates from KtyI-/-K14-cells, KtyI-/-mK17 cells and KtyI-/-mK17 cells overexpressing DPII-GFP 24h or 72h after Ca^2+^ switch. Stars are indicating endogenous DP1 and DP2 bands. (c’-c’’) Quantification of relative amounts of endogenous DP1 and DP2 protein levels from 3 different experiments. Tubulin was used as loading control, (mean+/- SEM, n=3, ns not significant; *p<0.05, **p<0.01, ***p<0.001, Student’s t-test.). (d-d’): Chemical crosslinking experiments with Sulfo-EGS show a significant reduction of Dsg3 cluster at the cell surface of KtyI-/-mK17 cells in comparison to KtyI-/-mK14 cells and restoration of Dsg3 clusters after overexpression of DPII-GFP in KtyI-/-mK17 cells. (d’) Quantification of relative amounts of Dsg3 oligomers. Tubulin was used as loading control, (mean+/- SEM, n=3, ns not significant; *p<0.05, **p<0.01, ***p<0.001, Student’s t-test.).


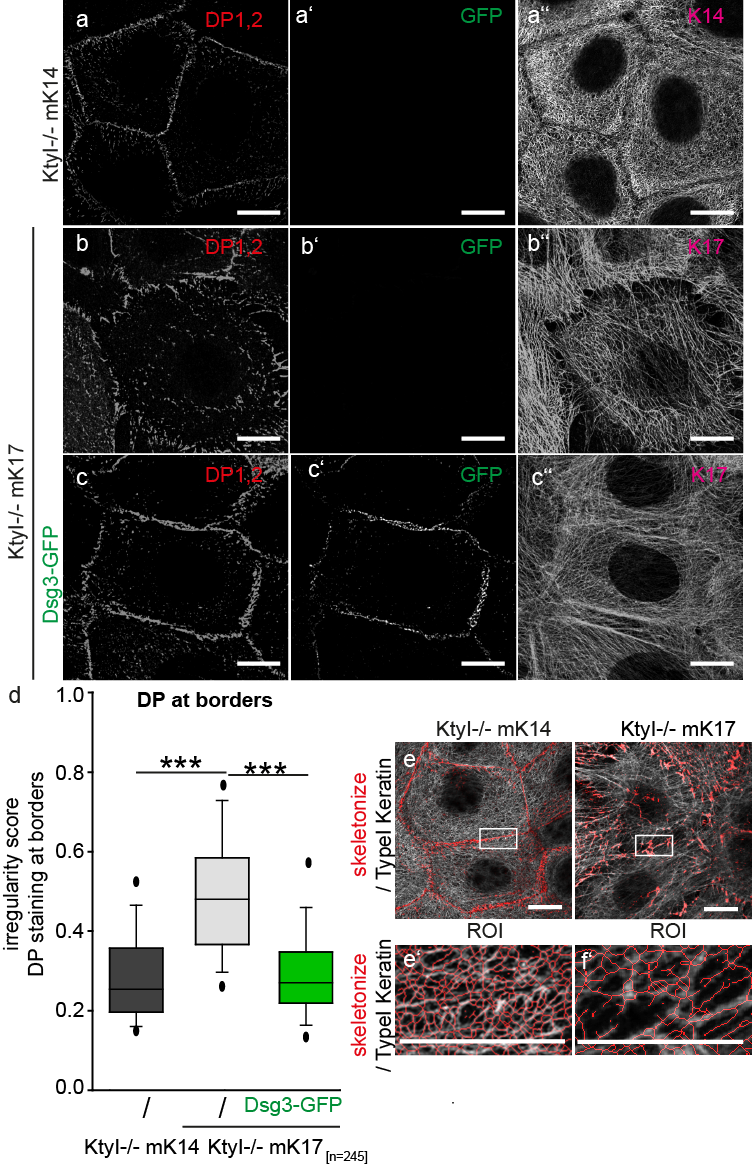


**Supplementary Figure 3 Overexpression of Dsg3-GFP in KtyI-/-mK17 cells restored the localization of desmoplakin at cell borders** (a-c’’) Representative immunostainings of DP and K14 or K17 in KtyI-/-mK14 and KtyI-/-mK17 cells 24h after increasing the Calcium concentration. Scale bar = 10µm. (d) Quantification of the irregularity score for DP at borders showing that the irregular DP localization at junctions in KtyI-/-mK17 cells was restored after Dsg3 overexpression. 245 cell-cell borders of 3 independent experiments were analyzed, ns not significant; *p<0.05, **p<0.01, ***p<0.001, Man-Whitney-Rank Sum test. (e-f) Representative confocal section of DP/keratin staining of KtyI-/-mK14 and KtyI-/-mK17 cells. (e’, f’) Overlay of skeletonized image with confocal image of K14 or K17 staining. Scale bar =10 µm.

**
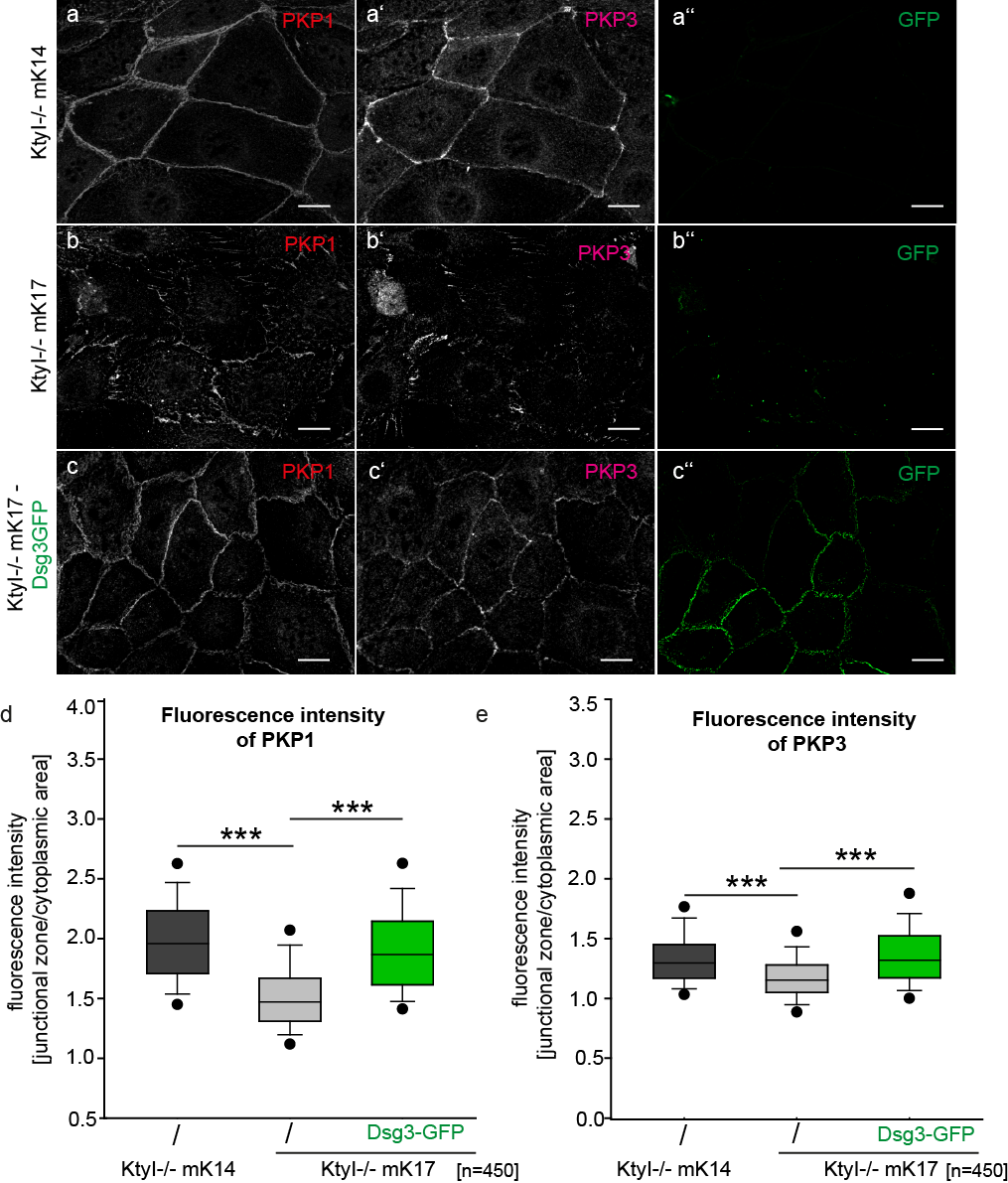
**

**Supplementary Figure 4 Overexpression of Dsg3-GFP in KtyI-/-mK17 cells restored the localization of PKP1 and 3 at cell borders** (a-c’’) Immunostaining of PKP1 and 3 in KtyI-/-mK14 and KtyI-/-mK17 cells 24h after Ca-induced junction formation. Scale bar = 10µm. (d-e): For quantification, the ratio of fluorescence intensity of PKP1 and 3 at junctions and in the cytoplasm was calculated using intensity profiles. 450 cell-cell borders of 3 independent experiments were analyzed, ns not significant; *p<0.05, **p<0.01, ***p<0.001, Man-Whitney-Rank Sum test.

**
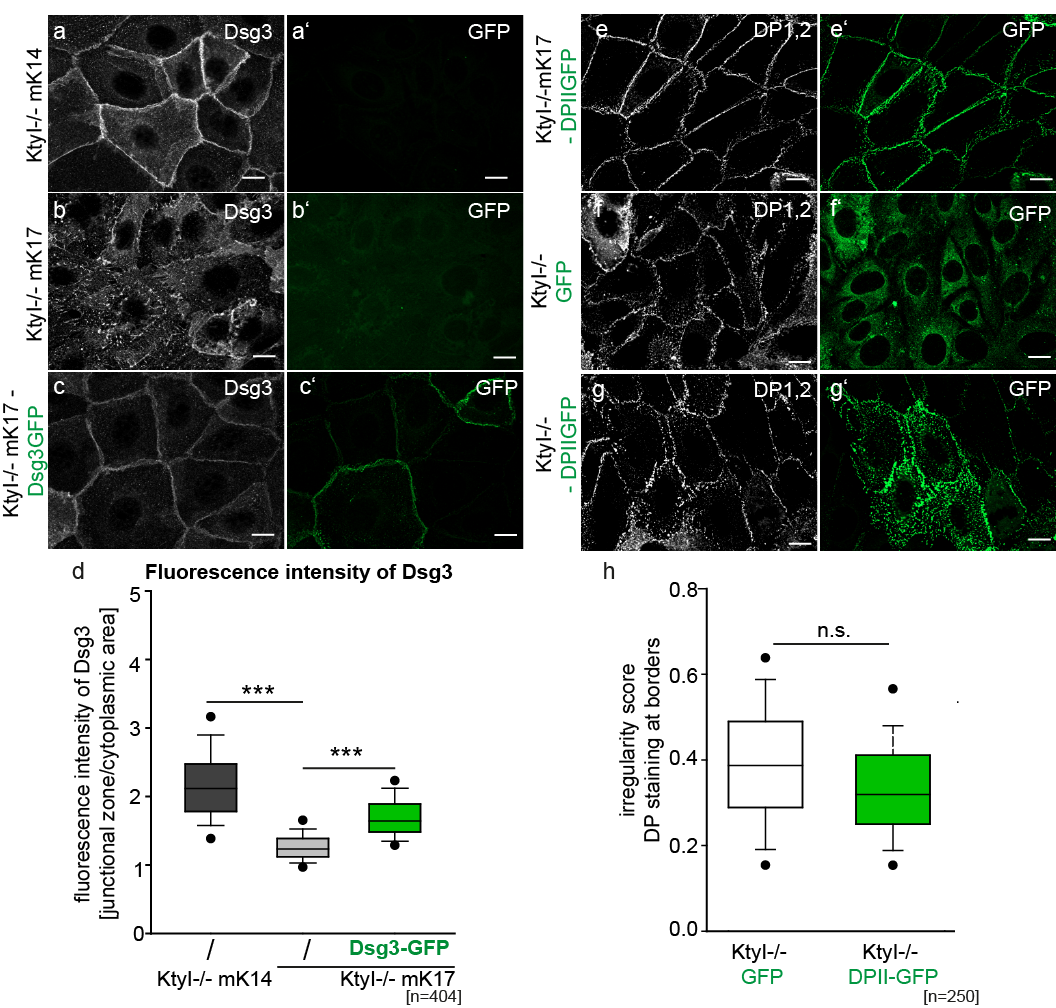
**

**Supplementary Figure 5 Overexpression of desmosomal components restore border localization of desmosomal proteins only in the presence of keratins** (a-c’): Immunostainings for Dsg3 show that overexpression of Dsg3-GFP rescued border localization of Dsg3 in KtyI-/-mK17 cells. (d) For quantification, the ratio of fluorescence intensity at junctions and in the cytoplasm was calculated using intensity profiles. 404 cell-cell borders of 3 independent experiments were analyzed, ns not significant; *p<0.05, **p<0.01, ***p<0.001, Man-Whitney-Rank Sum test. (e-g’): Immunostaining for DP in KtyI-/- mK17 cells overexpressing DPII-GFP and KtyI-/- cells overexpressing GFP or DPII-GFP show that overexpression of DPII-GFP in keratin-free keratinocytes (KtyI-/-) don’t restore the irregular localization of desmoplakin. Scale bar = 10µm. (h) Quantification of the irregularity score for DP at borders showed that the irregular DP localization at junctions in KtyI-/- cells expressing GFP or expressing DPII-GFP was not restored after DPII overexpression. 250 cell-cell borders of 3 independent experiments were analyzed, ns not significant; *p<0.05, **p<0.01, ***p<0.001, Man-Whitney-Rank Sum test.


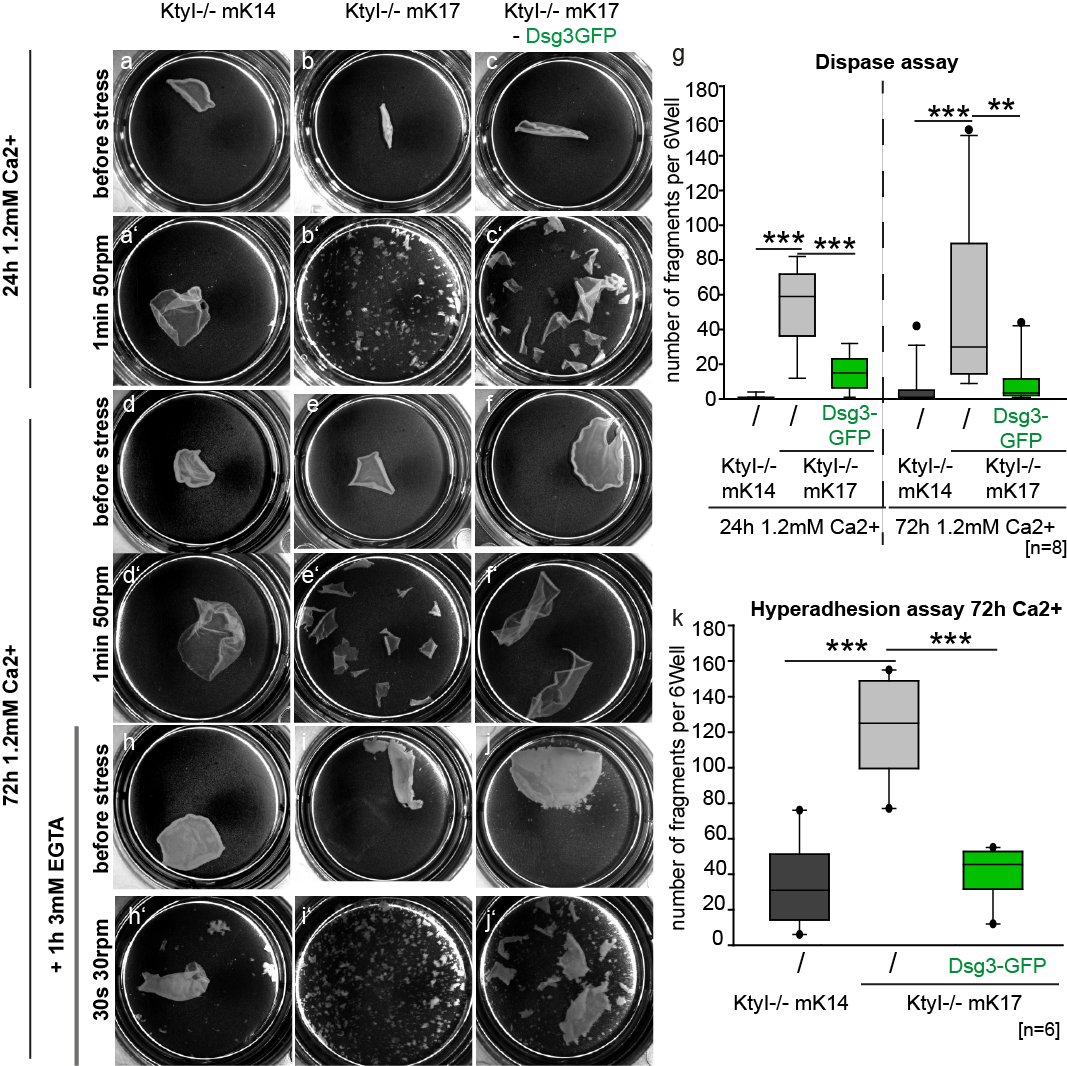


**Supplementary Figure 6 Overexpression of Dsg3-GFP in KtyI-/-mK17 cells stabilizes epithelial sheets** (a-k) Dispase and hyperadhesion assays show that overexpression of Dsg3GFP in KtyI-/-mK17 cells stabilized sheet stability and restored the formation of hyperadhesive desmosomes. (g and k) For quantification, number of fragments were counted. N=8 (g) n=6 (k); ns not significant; *p<0.05, **p<0.01, ***p<0.001, Student’s t-test.


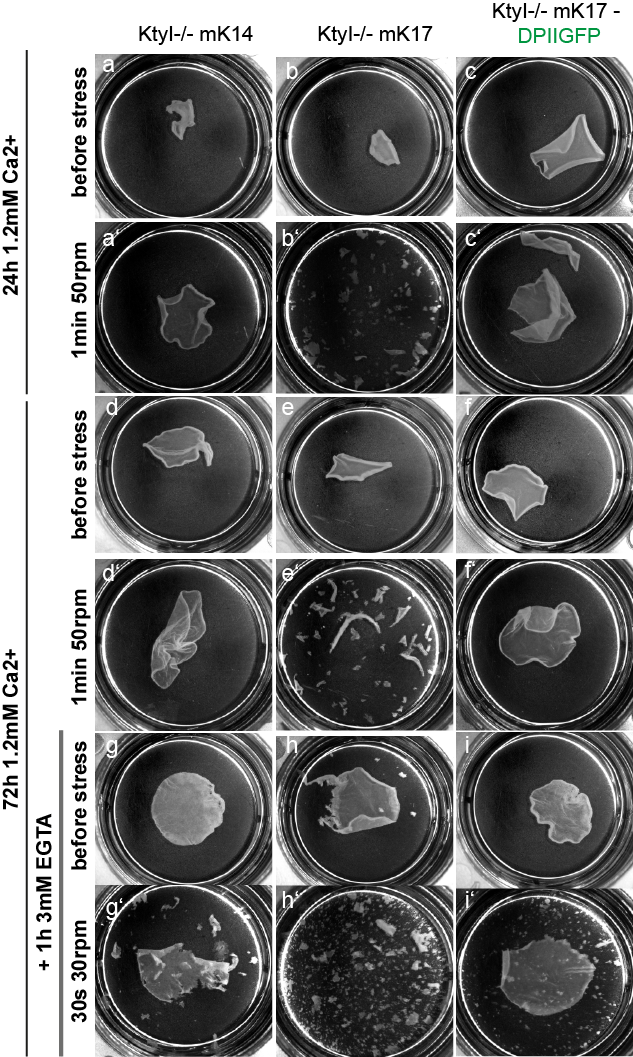


**Supplementary Figure 7 Overexpression of DPII-GFP in KtyI-/-mK17 cells stabilizes epithelial sheets** (a-i’) Representative images of the dispase and the hyperadhesion assays showing that overexpression of DPII-GFP in KtyI-/-mK17 cells stabilized epithelial sheets.


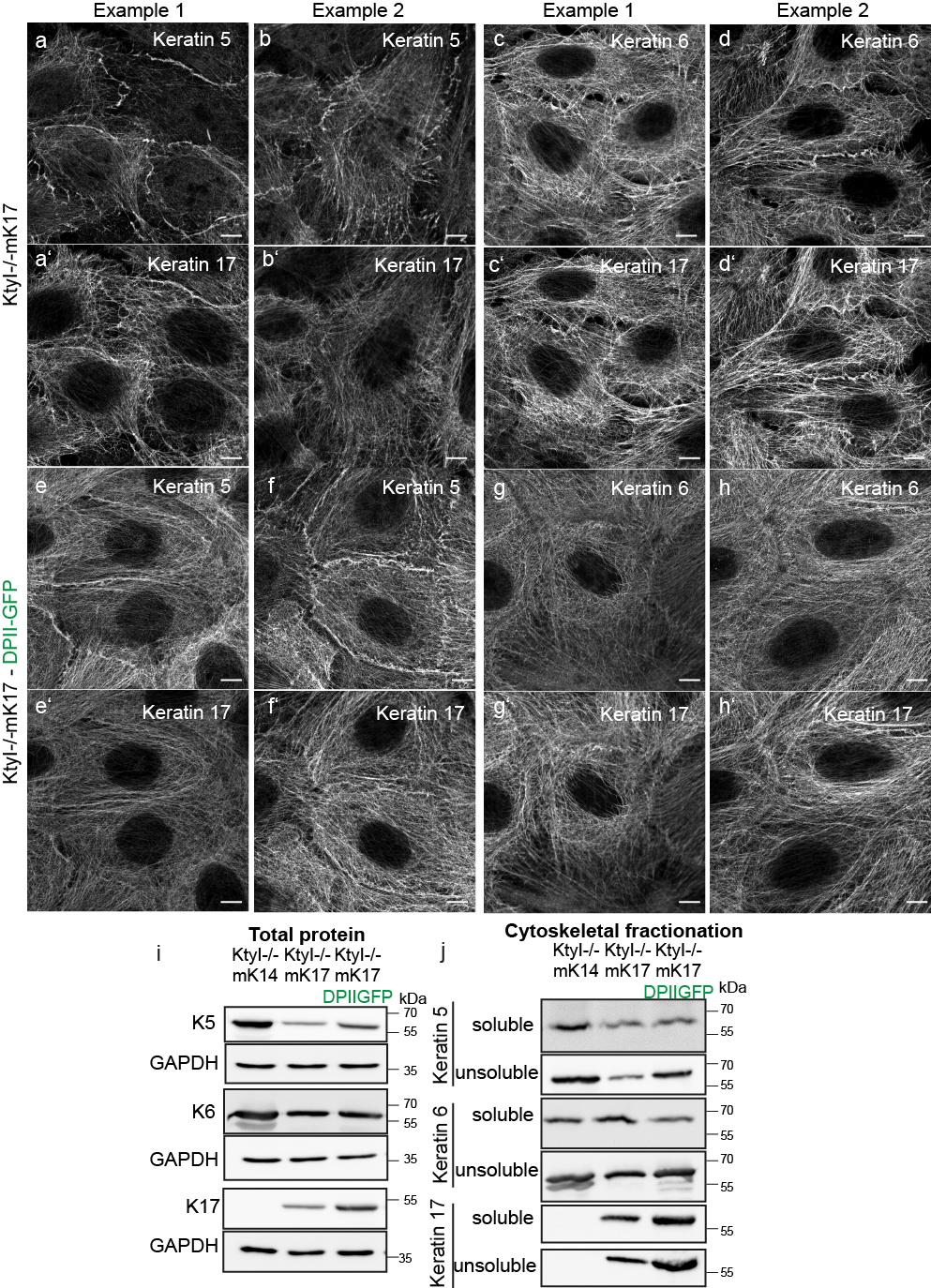


**Supplementary Figure 8 Overexpression of DPII-GFP increased the abundance of K5-containing filaments in K17 expressing cells:** (a-h’) Co-staining of K5/K17 or K6/K17 reveal altered keratin organization in K17 expressing cells after overexpression of DPII-GFP. Furthermore, K5-containing filaments increased after overexpression of DPII-GFP. Two representative images are shown for each cell line. (i) Representative western blots of total lysates for keratins 5, 6 and 17. GAPDH was used as loading control. (j) Representative cytoskeletal fractionation and subsequent WB of soluble and insoluble fractions. (k-m’): Representative images of DP-staining and GFP-fluorescence in KtyI-/-mK17 overexpressing DPII-GFP and keratin-free cells (KtyI-/-) expressing GFP alone or DPII-GFP. Scale bar: 10µm.
